# Diagnostic accuracy of serum and synovial biomarker thresholds for diagnosing periprosthetic joint infection: a QUADAS-C-guided systematic review and meta-analysis

**DOI:** 10.1186/s42836-026-00396-5

**Published:** 2026-05-11

**Authors:** Maria Sartori, Silvia Brogini, Deyanira Contartese, Francesco Rosa, Mirco Lo Presti, Maria Pia Neri, Matteo Romagnoli, Dante Dallari, Angelo Toscano, Stefano Zaffagnini, Milena Fini, Gianluca Giavaresi

**Affiliations:** 1 Surgical Sciences and Technologies, IRCCS Istituto Ortopedico Rizzoli, Via Di Barbiano 1/10, 40136 Bologna, Italy; 2 II Orthopaedic and Traumatologic Clinic, IRCCS Istituto Ortopedico Rizzoli, Via G.C. Pupilli 1, 40136 Bologna, Italy; 3 Orthopaedics and Traumatology, Istituto Ortopedico Rizzoli, Via Nazionale Ponente 5, 44011 Argenta, FE Italy; 4 Reconstructive Orthopaedic Surgery and Innovative Techniques-Musculoskeletal Tissue Bank, IRCCS Istituto Ortopedico Rizzoli, Via G.C. Pupilli 1, 40136 Bologna, Italy; 5 General Orthopaedics, IRCCS Istituto Ortopedico Rizzoli, Via G.C. Pupilli 1, 40136 Bologna, Italy; 6 Scientific Direction, IRCCS Istituto Ortopedico Rizzoli, Via Di Barbiano 1/10, 40136 Bologna, Italy

**Keywords:** Periprosthetic joint infection, Biomarkers, Synovial fluid, C-reactive protein, α-defensin, Diagnostic accuracy, D-dimer, Calprotectin

## Abstract

**Background:**

Periprosthetic joint infection (PJI) remains one of the most serious complications after joint arthroplasty, and accurate diagnosis continues to pose significant challenges. A reliable distinction between septic and aseptic failure is essential for appropriate surgical and antibiotic management. This systematic review and meta-analysis evaluate the most recent clinical evidence on the diagnostic accuracy, methodological quality, and standardization potential of established “classical” and emerging serum and synovial biomarkers for PJI diagnosis.

**Methods:**

This systematic review and meta-analysis were conducted in accordance with PRISMA-DTA guidelines, with PRISMA used as a general reporting framework. Selected items from the STARD checklist were used solely to inform data extraction and were not applied as a reporting guideline. Clinical studies published between January 1, 2020, and October 31, 2025, were identified through PubMed, Scopus, and Web of Science. Eligible studies reported sensitivity, specificity, and/or diagnostic thresholds for serum or synovial biomarkers based on MSIS, ICM, or EBJIS criteria for PJI definition. Study quality was assessed using the QUADAS-C tool, and a meta-analysis of chronic PJI cohorts was performed.

**Results:**

Twenty-six studies were included (8 serum, 18 synovial). Among serum biomarkers, C-reactive protein and erythrocyte sedimentation rate showed moderate diagnostic accuracy (Area Under the ROC curve [AUC] 0.82–0.90), while fibrinogen demonstrated comparable performance in selected cohorts. D-dimer and other coagulation indices showed variable results. Synovial biomarkers demonstrated superior diagnostic performance. α-defensin, D-lactate, calprotectin (CP), and neutrophil gelatinase-associated lipocalin achieved excellent accuracy (AUC > 0.90; sensitivity and specificity > 90%). Synovial white blood cell count (WBC) and polymorphonuclear percentage (PMN%) remained reliable and cost-effective. Meta-analysis confirmed high pooled diagnostic performance for WBC (0.88/0.97) and PMN% (0.84/0.97).

**Conclusions:**

Traditional serum biomarkers remain useful first-line diagnostic tools, whereas synovial biomarkers—particularly α-defensin, D-lactate, CP, and WBC indices—demonstrate superior diagnostic accuracy and may be especially valuable in culture-negative PJI. However, substantial heterogeneity and risk of bias identified by QUADAS-C—particularly in patient selection, reference standards, and threshold derivation—suggest that pooled estimates may represent optimistic upper-bound performance, limiting generalizability. Future diagnostic strategies should integrate multimodal biomarker panels with molecular diagnostics and artificial intelligence-based approaches to improve diagnostic precision and clinical applicability.

**Supplementary Information:**

The online version contains supplementary material available at 10.1186/s42836-026-00396-5.

## Introduction

Hip and knee joint replacements are among the most common and successful surgeries worldwide, representing a cornerstone of musculoskeletal medicine and driving biomedical innovation [1]. While primary joint arthroplasty usually provides clear benefits, revision surgeries are more complex, costly, and often result in poorer outcomes, particularly in older patients [2]. Periprosthetic joint infection (PJI) is the most severe complication, accounting for 28%–33% of early hip and knee revision procedures and typically occurring within two years of implantation [3]. Despite advances in surgical techniques, perioperative care, and biomaterials, PJI remains a major clinical and economic burden. Diagnosing PJI is challenging due to the heterogeneous clinical and laboratory presentations that may mimic other conditions. Timely detection is crucial, as treatment strategies depend on the patient’s clinical scenario. To address this issue, international groups have developed diagnostic criteria [4, 5], including those proposed by the Musculoskeletal Infection Society (MSIS) and later revised by the 2018 International Consensus Meeting (ICM). These criteria combine traditional serum and synovial tests, such as C-reactive protein (CRP), white blood cell count (WBC), and polymorphonuclear percentage (PMN%), with “newer” biomarkers, including leukocyte esterase (LE), D-dimer, and alpha-defensin [6–9].

In 2021, the European Bone and Joint Infection Society (EBJIS) further refined these criteria by updating biomarker cut-offs, incorporating techniques such as sonication and nuclear imaging, and emphasizing α-defensin testing through immunoassay or lateral flow methods [10]. Despite these updates, the diagnosis of PJI remains uncertain, with false-negative rates reported in up to 34% of cases [11, 12]. Only a limited number of new biomarkers have been formally incorporated into diagnostic criteria, highlighting the ongoing need for markers that are specific, sensitive, cost-effective, and clinically reliable [6, 7]. Currently, variability in diagnostic thresholds and testing methodologies limits their widespread adoption, underscoring the need for robust evidence evaluating their diagnostic accuracy.

In this scenario, this systematic review and meta-analysis aimed to evaluate the diagnostic accuracy of serum and synovial biomarkers for PJI based on evidence published between 2020 and 2025. Particular attention was given to sensitivity, specificity, and diagnostic thresholds across different biomarkers, while methodological quality was assessed using the Quality Assessment of Diagnostic Accuracy Studies Comparative (QUADAS-C) tool. By synthesizing the most recent clinical evidence, this study aims to clarify the relative diagnostic value of traditional and emerging biomarkers and their potential role within contemporary diagnostic algorithms for PJI.

## Materials and methods

The current systematic review was conducted in accordance with the PRISMA-DTA (Preferred Reporting Items for Systematic Reviews and Meta-Analyses of Diagnostic Test Accuracy Studies) guidelines, using the PRISMA (Preferred Reporting Items for Systematic Reviews and Meta-Analyses) framework as a general reporting standard [13]. Selected items from the STARD (Standards for Reporting of Diagnostic Accuracy Studies) checklist were used to guide data extraction and to ensure completeness in reporting diagnostic accuracy, rather than being applied as a formal reporting guideline [14]. The review protocol was registered in PROSPERO (CRD420251170688). The search strategy was designed to identify studies evaluating serum and synovial biomarkers for the diagnosis of PJI. Studies published between January 1, 2020, and October 31, 2025, were considered eligible. Study selection, data extraction, and data synthesis were conducted in accordance with PRISMA-DTA recommendations.

### Search strategy

In the first phase, an appropriate PICO question was formulated to identify all clinical studies (Population), evaluating the diagnostic accuracy (Outcome) of novel biomarkers (Intervention), in terms of sensitivity, specificity, and predictive value, as diagnostic tools for PJI, compared with established reference criteria (Comparison). To address this objective, we screened articles indexed in three major electronic databases, PubMed, Scopus, and Web of Science, covering the period from January 1, 2020, to October 31, 2025. To minimize the risk of missing any relevant study, the search strategy was deliberately broad, combining Boolean operators with Medical Subject Headings (MeSH terms) and free-text keywords related to PJI and diagnostic biomarkers. The final search query was as follows: (“periprosthetic joint infection” OR “PJI” OR “prosthetic joint infection” OR “infected arthroplasty”) AND (“biomarker” OR “serum marker” OR “synovial fluid marker” OR “diagnostic marker”) AND (“diagnosis” OR “diagnostic accuracy” OR “sensitivity” OR “specificity” OR “ROC”). Additional eligible studies were identified through manual screening of the reference lists of the included articles.

### Inclusion and exclusion criteria

We included all eligible clinical studies, prospective, retrospective, or cross-sectional, that focused on the diagnostic accuracy of serum and/or synovial biomarkers in patients undergoing revision surgery after hip and/or knee arthroplasty for suspected chronic PJI. Studies were considered eligible if they assessed at least one serum or synovial biomarker for PJI diagnosis and explicitly reported data on sensitivity, specificity, or predictive values of the investigated biomarker. To ensure methodological rigor, only studies employing a clinical reference standard for PJI diagnosis, as defined by the MSIS, ICM, and/or EBJIS criteria, were included. Excluded case reports, letters, editorials, reviews, conference abstracts, and preclinical studies, as well as studies not focused on diagnostic testing or those assessing biomarkers in contexts unrelated to PJI (e.g., osteomyelitis). Studies that did not report sensitivity and specificity values were also excluded. Only full-text articles published in English were included.

**Fig. 1:**
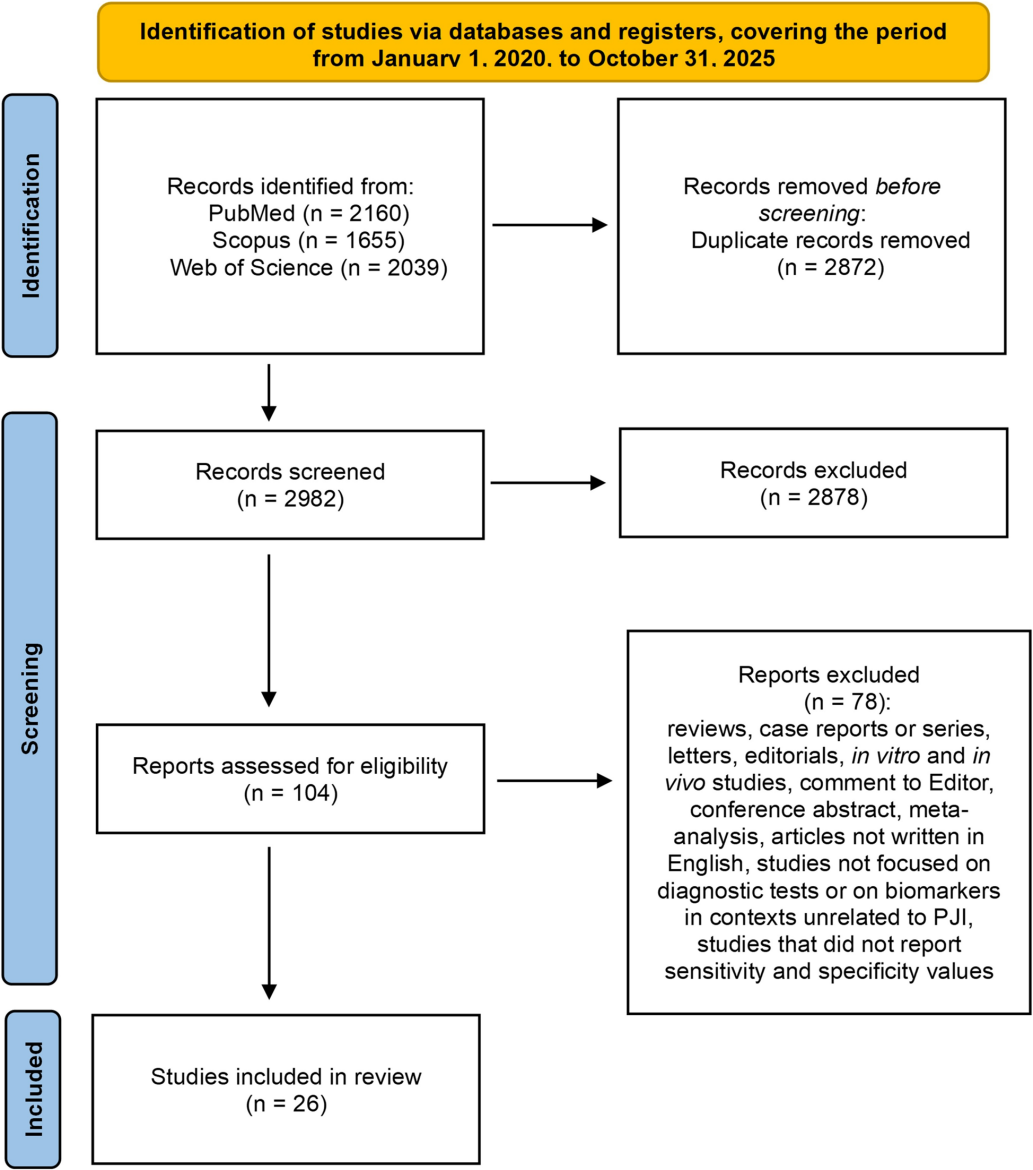
PRISMA flow diagram illustrates the systematic review process, including details of database searches, number of abstracts screened, full-text articles assessed for eligibility, and final studies included

All records were initially downloaded as titles and abstracts and imported into Rayyan [15] to remove duplicates. Screening was then performed independently by two reviewers (SB, GG) according to the predefined inclusion and exclusion criteria (Fig. 1).

### Data extraction

Each selected study was independently evaluated twice by four reviewers (DC, SB, MS, FR), who extracted data using a customized protocol including the following variables: study type, country, setting, study period, inclusion and exclusion criteria, number of patient enrolled and analyzed (N), mean age, percentage of female patients (%F), joint site (Hip/Knee [N]), number of infected joints, details of the index test (assay, brand, sample handling, blinding, timing), comparators, flow and timing (verification and exclusions), and microbiological findings. All extracted information was summarized in dedicated summary tables (Tables S1 and S2, Supplementary Materials). An additional table was created to report diagnostic accuracy parameters, including sensitivity, specificity, AUC with respective cut-off values, and cut-off reference standards (Table S3, Supplementary Materials). Both established and novel biomarkers were considered. In cases of disagreement, a fifth reviewer (GG) resolved the discrepancies through discussion and consensus. Finally, the extracted data were categorized into two main domains: serum biomarkers and synovial biomarkers.

### Quality assessment

The quality of each included study was assessed using the QUADAS-C tool [16]. Derived from QUADAS-2, QUADAS-C has been specifically adapted to address methodological challenges in the direct comparison of diagnostic tests, with particular focus on patient selection, test execution, comparability, and statistical analysis. The use of QUADAS-C allows for a systematic and standardized evaluation of study quality, thereby improving the reliability of the evidence synthesis and supporting the development of more robust clinical recommendations.

### Data analysis

A diagnostic accuracy meta-analysis was performed using 2 × 2 contingency tables at fixed thresholds (ICM or manufacturer-defined). When raw count data were not directly reported, 2 × 2 tables were reconstructed from reported sensitivity, specificity, and sample size using deterministic calculations, minimizing error. Sensitivity and specificity were calculated with exact Clopper–Pearson confidence intervals (CIs). *Z*ero cells were handled using a 0.5 continuity correction prior to logit transformation. For tests or platforms evaluated in ≥ 3 independent studies, a bivariate random-effects model (Reitsma method, logit scale) was fitted to jointly estimate pooled sensitivity and specificity with 95% CIs; where appropriate, 95% prediction intervals were also reported. When applicable, hip and knee cohorts were analyzed separately. In sensitivity analyses, joint-level data pooled within studies were aggregated by summing true positive (TP), false negative (FN), false positive (FP), and true negative (TN). Between-study heterogeneity was assessed using the variance components of the random-effects model and summarized using I^2^ statistics for sensitivity and specificity. When the number of studies was ≤ 2, results were synthesized narratively and, where informative, by simple pooling of counts (sensitivity = ΣTP/Σ[TP + FN]; specificity = ΣTN/Σ[TN + FP]). Likelihood ratios (LRs) were derived from pooled sensitivity and specificity; study-level LRs were calculated using exact binomial methods. Sensitivity analyses were informed by QUADAS-C, excluding studies at high risk of bias or with potential incorporation bias. All analyses were performed using R software [17], with visualization conducted using ggplot2 [18]. A complete dataset of TP, FP, FN, and TN for each included study is provided in the Supplementary Materials (Table S4), with explicit indication of whether data were directly reported or reconstructed. Reconstruction methods are fully detailed in the Supplementary Materials.

## Results

### Serum biomarkers

#### Study selection and characteristics

Eight studies investigating serum biomarkers were included in this systematic review: two prospective studies [19, 20] and six retrospective studies [21–26]. All studies were conducted at a single center. The main demographic and study design characteristics are summarized in Table S1 (Supplementary Materials).

Seven of the eight studies included both hip and knee arthroplasties [19–22, 24], although separate data for each joint were not always reported. One study included only hip arthroplasties [23]. Among studies reporting joint distribution, several described a higher number of hip than knee infections, whereas in the largest cohort, hip and knee PJIs were nearly balanced (215 hips vs. 220 knees within a revision population of 542 hips and 299 knees) [21]. Two studies that included both hip and knee revisions did not specify the distribution of infections between the joints [25, 26].

Most studies used the ICM or MSIS criteria as the reference standard for PJI diagnosis, while one study applied the Infectious Diseases Society of America (IDSA) criteria [20]. The extracted data on serum biomarkers are summarized in Table S1 (Supplementary Materials).

The investigated biomarkers (index tests) varied across studies but mainly focused on fibrinolytic and inflammatory markers. D-dimer was evaluated in four studies [20, 22, 24, 25] and fibrinogen in two [24, 26], usually alongside conventional inflammatory markers such as CRP and erythrocyte sedimentation rate (ESR), which served as comparators in most cohorts. Other biomarkers included interleukin (IL)-6 [26] and serum procalcitonin (PCT) [19].

Some studies also assessed coagulation-related parameters, such as fibrin degradation products (FDP), platelet count (PC), and platelet volume ratio (PVR) [22], as well as blood cell-derived ratios including neutrophil-to-lymphocyte ratio (NLR), platelet-to-lymphocyte ratio (PLR), and monocyte-to-lymphocyte ratio (MLR) [26]. Additionally, ratio-based inflammatory indices were evaluated, particularly the CRP/hemoglobin ratio (CHR) and several CRP- or ESR-derived combinations (e.g., CRP + ESR, CRP/albumin, CRP/[Hb + Alb], ESR/albumin, ESR/Hb) [21, 24].

### Synovial biomarkers

#### Study selection and characteristics

Eighteen studies investigated synovial fluid biomarkers for the diagnosis of PJI (Table S2, Supplementary Materials). Most were prospective (14/18), while four were retrospective. Prospective studies were predominantly single-center (12/14); the only multicenter studies were conducted by Fuchs et al. and Güneş et al., who evaluated D-lactate and D-dimer, respectively [27, 28]. All retrospective studies [29–32] were conducted at single centers.

Most cohorts included both hip and knee arthroplasties, whereas two studies focused exclusively on knees [32, 33]. The prevalence of PJI ranged from 22% [34] to 64% [35], with approximately 1,090 infections among about 2,650 arthroplasties overall. Knee infections predominated in most cohorts reporting joint-level data—often accounting for around two-thirds of PJIs—and this pattern was reinforced by the knee-only series [32, 33]. Hip predominance was uncommon and mainly observed in smaller cohorts (e.g., 14/26 in Lazic et al. [36] and 10/14 in Mihalič et al. [34]), whereas several studies showed a more balanced distribution between joints (e.g., Qin et al., 6/11 [37]; Suren et al., 11/25 [38]).

All studies used reference standards based on ICM or MSIS criteria, with two also incorporating the 2021 EBJIS recommendations [31, 36]. The evaluated synovial biomarkers showed considerable heterogeneity and included cellular indices, protein biomarkers, enzymatic or metabolic analytes, and rapid point-of-care assays. Core cellular parameters—synovial WBC and PMN%—were frequently assessed and compared with contemporary diagnostic definitions (MSIS/ICM/EBJIS) [32, 34, 39]. Synovial CRP was studied both as a standalone test and in combination with serum CRP [29, 39]. Alpha-defensin was evaluated using both Enzyme-Linked Immunosorbent Assay (ELISA) on frozen samples and lateral-flow tests on fresh synovial fluid, often compared with LE strips and synovial cell counts [33, 40]. Calprotectin (CP) lateral-flow testing (Lyfstone) was assessed in two distinct clinical settings: Intra-operative testing during revision for periprosthetic fractures [36] and routine revision arthroplasty [38]. Synovial PCT was evaluated in one study but did not reliably differentiate PJI from aseptic loosening [19].

Emerging biomarkers included D-lactate measured by colorimetric assay or disposable strip tests [27, 41]; Neutrophil Gelatinase Associated Lipocalin (NGAL), quantified by ELISA on stored samples [35]; and cytokine panels including IL-1β, IL-2, IL-4, IL-6, IL-8, IL-10, IL-12, and IL-17 [37]. Several studies also emphasized joint- and chronicity-specific thresholds. For example, synovial CRP cut-offs were adapted for hip versus knee arthroplasties and for acute versus chronic infections [29, 42], while the absolute PMN count (Absolute Polymorphonuclear count [APMN] = WBC × PMN%/100) was validated using joint- and definition-specific decision thresholds for both EBJIS and ICM 2018 criteria [31]. Large registry-based cohorts using same-encounter sampling further confirmed the diagnostic value of synovial CRP, WBC/PMN, and APMN, highlighting how thresholds may vary depending on the applied reference standard [30, 32].

### Quality assessment (QUADAS-C)

Methodological quality and applicability were assessed using the QUADAS-C tool across all identified study-comparison pairs (44 comparisons from 26 studies; 18 prospective and 10 retrospective non-cross-sectional studies). A focused synthesis was subsequently performed on a subset of chronic PJI studies used for quantitative pooling [29, 33, 38, 43].

Eligibility for the chronic meta-analysis required: (1) an explicitly chronic population, defined as ≥ 90 days after implantation or a closely comparable definition; (2) availability or derivability of complete 2 × 2 diagnostic data at defined thresholds; (3) use of a consistent and defensible reference standard. When raw counts were unavailable, 2 × 2 tables were reconstructed from published sensitivity and specificity values (and confidence intervals, when available), with minimal rounding error. Potential incorporation bias and data-driven thresholds were flagged within the QUADAS-C assessment. Restricting the analysis to chronic infections helped reduce spectrum effects, which in mixed acute-chronic cohorts may artificially increase the apparent sensitivity of synovial tests.

**Fig. 2:**
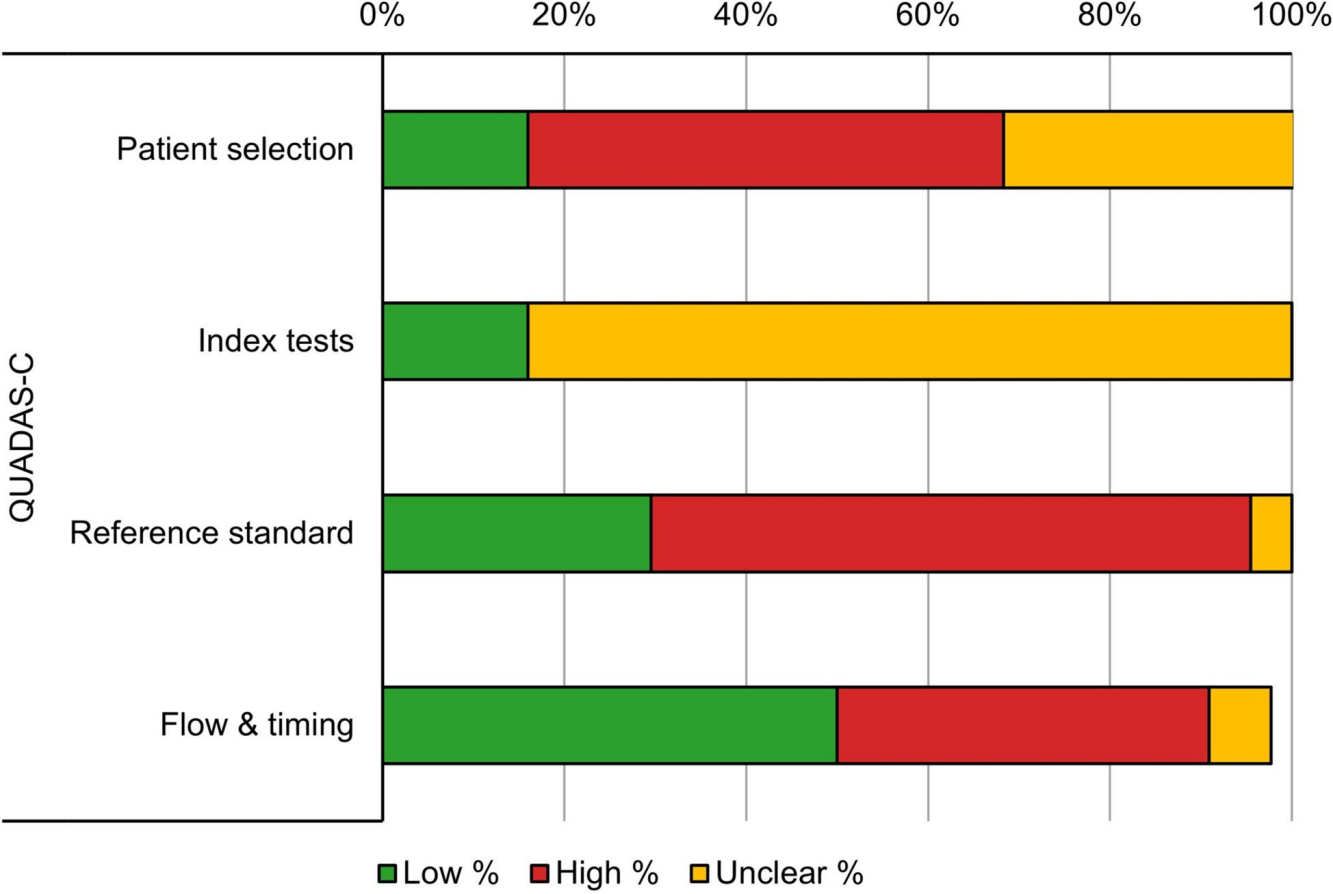
QUADAS C summary across all comparisons (*n* = 44). Stacked bars show the proportion of Low (green), High (red), and Unclear (yellow) comparative risk of bias judgments by domain: Patient selection—Low 16%, High 52%, Unclear 32%; Index tests—Low 16%, High 0%, Unclear 84%; Reference standard—Low 30%, High 66%, Unclear 5%; Flow & timing—Low 50%, High 41%, Unclear 7%

Across all comparisons (Fig. 2), QUADAS-C revealed substantial concerns rather than predominantly low comparative risk of bias: Patient selection—Low 16%, High 52%, Unclear 32%; Index tests—Low 16%, High 0%, Unclear 84%; Reference standard—Low 30%, High 66%, Unclear 5%; Flow & timing—Low 50%, High 41%, Unclear 7%.

High or unclear risk most frequently resulted from incorporation of index tests into the reference standard (often favoring synovial cell count or synovial CRP), differences in timing or specimen processing between tests (e.g., pre-operative blood vs. intra-operative synovial fluid, fresh vs. frozen aliquots), incomplete or differential verification, and insufficient reporting of index test procedures or independence.

Study-level extraction further highlighted several sources of bias. Thresholds for index tests were often optimized using ROC or Youden analysis within the same dataset rather than prespecified, potentially inflating diagnostic accuracy. Pre-analytical handling also varied, including the use of fresh versus frozen/thawed aliquots, selective centrifugation of bloody samples, and device-dependent reading. LE testing could not be performed in approximately one-third of aspirations due to hemarthrosis. Some comparisons included reimplantation or second-stage samples among the non-infected controls.

Patient populations were frequently restricted by exclusions (e.g., inflammatory comorbidities, other infections, or recent antibiotic use) or by the use of non-comparable control groups such as primary osteoarthritis. Certain cohorts also represented highly selected clinical settings, including metal-on-metal or rotating hinge knees, periprosthetic fractures, and salvage total hip arthroplasty (THA).

Reference standards commonly relied on composite ICM/MSIS/EBJIS definitions incorporating CRP/ESR or synovial WBC/PMN, and occasionally synovial CRP or α-defensin, while some studies applied modified institutional criteria or post-operative data. Partial or differential verification occurred when tests were infeasible or not performed in all participants, and reporting of blinding procedures and handling of indeterminate results was frequently incomplete.

### Diagnostic accuracy summary

Table S3 (Supplementary Materials) summarizes the diagnostic performance of the evaluated index tests in each study, including AUC with 95% CI, diagnostic cut-offs, sensitivity and specificity, reference standards, and key methodological notes.

The diagnostic performance of serum biomarkers was heterogeneous. Conventional inflammatory markers, particularly CRP and ESR, remained the most reliable and widely validated systemic indicators, with sensitivity and specificity frequently exceeding 80% across cohorts, including culture-negative infections [20, 22]. Their stability supports continued integration into diagnostic algorithms, even in polymicrobial or culture-negative scenarios.

In contrast, coagulation-related markers such as D-dimer and FDP showed variable and generally lower accuracy, reflecting susceptibility to post-operative and systemic inflammatory fluctuations. For example, Chen et al. reported the lowest diagnostic performance for serum D-dimer (AUC 0.57) compared with CRP (AUC 0.88) and ESR (AUC 0.82), which remained reliable across both culture-positive and culture-negative subgroups [20, 22].

Among emerging serum markers, fibrinogen showed encouraging results—approaching the accuracy of CRP/ESR, particularly when combined with cellular ratios (AUC > 0.90)—whereas PCT demonstrated limited diagnostic value (AUC < 0.60). Ratio-based inflammatory indices also showed promising performance: Aimaiti et al. [21] reported that the CHR, C-reactive protein to Albumin Ratio (CAR), and C-reactive protein to Hemoglobin-Albumin Ratio (C/HAR) achieved the highest accuracies among serum tests (AUC 0.872–0.873). At a CHR cut-off of 0.078, sensitivity and specificity were 81.3% and 83.9%, respectively, slightly surpassing CRP alone (AUC 0.860; 11.05 mg/L). These findings suggest that systemic biomarkers primarily reflect the intensity of the host inflammatory response rather than pathogen burden or culture positivity. Overall, serum markers remain valuable systemic indicators but do not match the diagnostic specificity of modern synovial assays, supporting their complementary role within integrated diagnostic pathways.

Synovial biomarkers generally demonstrated higher diagnostic accuracy. Baek et al. reported that synovial α-defensin achieved the strongest performance (AUC 0.93; sensitivity 94.4%; specificity 89.5%), outperforming serum ESR and CRP [33]. Synovial WBC count and PMN% provided excellent discrimination across several cohorts and remain reliable, accessible reference tests [27, 29, 32, 33, 39, 41], with combined sensitivities of 94.4–100% and specificities of 92.9–100%. Baker et al. confirmed excellent performance for synovial CRP (AUC 0.951; sensitivity 74.2%; specificity 98.0%), which consistently outperformed serum CRP and benefited from joint- and chronicity-specific thresholds [29, 30]. Across 616 hip and knee revisions, Sebastian et al. validated joint-specific thresholds for the synovial absolute PMN count (APMN = WBC × PMN%/100), demonstrating high accuracy independent of the applied diagnostic definition. After excluding acute procedures, thresholds proposed by EBJIS and ICM 2018 remained stable. Importantly, APMN showed higher sensitivity and negative predictive value than α-defensin and remained informative in borderline diagnostic scenarios [31].

Rapid and lateral-flow assays remain central to point-of-care strategies: α-defensin and LE strips can be read directly on fresh aspirates with immediate turnaround. However, LE testing may be technically infeasible in the presence of macroscopic hemarthrosis unless centrifugation is performed; when both assays are applicable, high concordance has been reported [40]. Haertlé et al. found that adding a glucose strip to LE provided minimal additional diagnostic value [44].

Additional synovial biomarkers further support the role of synovial testing. Synovial NGAL demonstrated high diagnostic accuracy and remained unaffected by prior antibiotic exposure [35]. CP lateral-flow assays showed reliable performance in both complex revisions and periprosthetic fractures, with optimal thresholds around 76–86 mg/L and sensitivity/specificity approximating 0.90–0.95 [36, 38]. Synovial D-dimer also showed excellent discrimination (AUC 0.992; sensitivity 100%; specificity 94% at a cut-off of 236,804 ng/mL), outperforming serum CRP and plasma D-dimer and showing similar accuracy to ESR [28].

Overall, modern synovial biomarkers, particularly α-defensin, D-lactate, and APMN, demonstrate high diagnostic accuracy (often AUC > 0.90) and perform well even in culture-negative infections, supporting their integration into multimodal diagnostic algorithms. Synovial WBC and PMN% remain reliable, cost-effective anchors, while synovial CRP provides a bridge between traditional inflammatory and molecular assays. Emerging markers, such as NGAL and cytokines (e.g., IL-6, IL-1β, IL-17), show promising but context-dependent performance (AUC > 0.85), suggesting a complementary role. In contrast, platelet count-based indices appear to have limited diagnostic utility (Table 1).

**Table 1:** Summary of diagnostic performance of major biomarkers

Biomarker	Type	Number of studies	Sensitivity range	Specificity range	AUC range
CRP	Serum	6	0.70–0.90	0.75–0.92	0.82–0.90
ESR	Serum	5	0.68–0.88	0.70–0.89	0.80–0.88
D-dimer	Serum	4	0.45–0.85	0.50–0.90	0.57–0.83
α-defensin	Synovial	4	0.90–0.96	0.85–0.97	0.90–0.95
Calprotectin	Synovial	3	0.85–0.94	0.88–0.95	0.90–0.96
D-lactate	Synovial	3	0.87–0.95	0.90–0.97	0.92–0.98
WBC	Synovial	6	0.85–0.95	0.92–0.98	> 0.90
PMN%	Synovial	5	0.80–0.92	0.90–0.97	> 0.88

### Meta-analysis

The meta-analysis was restricted to chronic cohorts (≥ 90 days post-implantation or equivalent definitions) with complete or reconstructible 2 × 2 diagnostic data at prespecified thresholds [36, 37, 45, 46]. When raw counts were unavailable, 2 × 2 tables were reconstructed from published sensitivity and specificity values (and confidence intervals, when reported), introducing only minimal rounding error. Pooling decisions were guided by study-level characteristics (Table S4, Supplementary Materials).

Several head-to-head comparisons used reference standards incorporating synovial parameters (e.g., synovial WBC/PMN within MSIS/ICM definitions), potentially compromising independence when comparing synovial and serum biomarkers and raising applicability concerns for the reference standard. Thresholds were also handled heterogeneously: in Baker et al., thresholds were largely prespecified (synovial CRP 6.9 mg/L, serum CRP 1.0 mg/dL, ESR 30 mm/h, synovial PMN% 80%, synovial WBC 3,000/µL) [29], whereas Baek et al. applied a non-prespecified α-defensin ELISA cut-off and did not report WBC/PMN thresholds [33]. Similarly, Sebastian et al. did not prespecify cut-offs for synovial or serum CRP, synovial WBC, or synovial PMN% [30, 31].

In most studies, 2 × 2 data were derived rather than directly reported (with few exceptions, such as Ackmann et al. [43]), introducing potential rounding or reconstruction error. Laboratory platforms also differed across studies (e.g., ELISA vs. lateral flow for α-defensin; Sysmex XN-550 for synovial WBC/PMN; Abbott Architect c4000 for serum CRP; immunoturbidimetry for serum calprotectin), which may influence absolute values without necessarily affecting relative diagnostic performance. Given these limitations—and generally acceptable flow and timing—comparisons between synovial and serum tests were interpreted cautiously, emphasizing absolute performance rather than direct superiority.

**Fig. 3:**
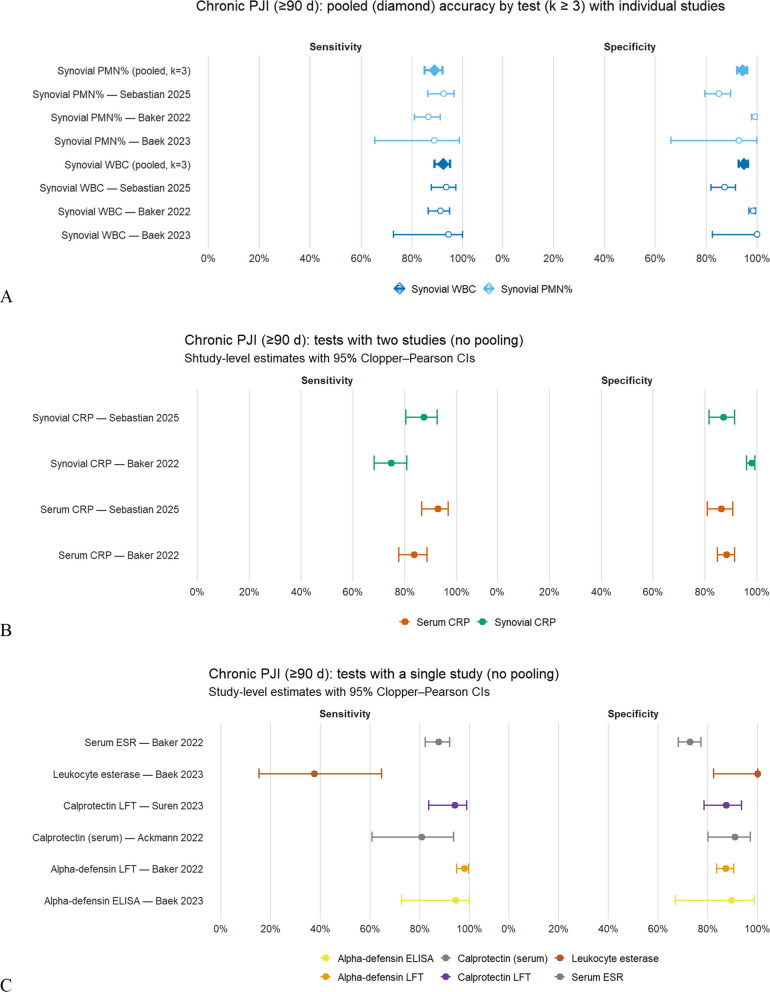
Diagnostic accuracy of tests for chronic peri-prosthetic joint infection (≥ 90 days). (**A**) Forest plots for tests with k ≥ 3: summary estimates (diamonds) from the bivariate random effects model (Reitsma, logit) with 95% CIs; individual study estimates with exact 95% CIs. (**B**) Tests with k = 2: study level estimates only (no pooling), with 95% Clopper–Pearson CIs. (**C**) Tests with k = 1: single study estimates (no pooling), with 95% Clopper–Pearson CIs. Sensitivity is on the left and specificity on the right in each panel

Figure 3A (chronic cohorts ≥ 90 days) demonstrates high pooled diagnostic accuracy for synovial cell counts across three paired studies (k = 3). For synovial WBC, random-effects pooling yielded: sensitivity of 0.88 (95% CI, 0.78–0.94; prediction interval 0.67–0.97); specificity of 0.97 (95% CI, 0.94–0.99; prediction interval 0.91–0.99); positive likelihood ratio (LR +) of ~ 35 (range 19–62); negative likelihood ratio (LR −) of ~ 0.10 (0.06–0.19).

Heterogeneity was moderate to high for sensitivity (I^2^ ≈ 73%) and lower for specificity (I^2^ ≈ 47%). Synovial PMN% showed slightly lower sensitivity but similar specificity: sensitivity of 0.84 (95% CI, 0.75–0.89; prediction interval 0.69–0.92); specificity of 0.97 (95% CI, 0.89–0.99; prediction interval 0.74–0.998); LR + of ~ 28 (range 12–114); LR − of ~ 0.16 (0.12–0.23).

Heterogeneity was moderate for sensitivity (I^2^ ≈ 57%) and higher for specificity (I^2^ ≈ 77%). Across studies, synovial WBC consistently showed slightly higher sensitivity than PMN% (+ 0.04 to + 0.06), while specificity was similar between the two tests. Leave-one-out analyses did not materially alter these results.

For biomarkers reported in only two studies (Fig. 3B; not pooled), synovial CRP demonstrated moderate to high sensitivity (≈0.75–0.90) and higher specificity (≈0.92–0.98), whereas serum CRP showed similar sensitivity but lower specificity (≈0.86–0.91).

Findings from single-study analyses (Fig. 3C) were broadly consistent with these patterns: α-defensin (lateral-flow/ELISA) and synovial CP demonstrated high sensitivities (> 0.90) with method-dependent specificity (≈0.85–0.97); LE strips showed low sensitivity (~ 0.35–0.40) despite near-perfect specificity; ESR demonstrated high sensitivity (~ 0.90–0.95) but only moderate specificity; Serum CP showed comparatively modest diagnostic performance.

## Discussion

### Principal findings

The use of biomarkers with limited diagnostic accuracy may complicate the identification of PJI, potentially delaying appropriate treatment. This review aimed to evaluate the diagnostic performance of both traditional and emerging serum and synovial biomarkers by synthesizing evidence from recent clinical studies.

Among serum biomarkers, conventional systemic markers, particularly CRP and ESR, remain the most reliable, cost-effective, and widely accessible tests. These markers offer a reasonable balance between sensitivity and specificity and are essential for initial screening within multimodal diagnostic algorithms. In contrast, newer candidates, such as D-dimer and other coagulation-related parameters, although pathophysiologically appealing, have not consistently demonstrated superior diagnostic accuracy and often exhibit greater variability in discriminatory performance. Fibrinogen, however, consistently emerged as a promising adjunct, with diagnostic performance comparable to CRP and ESR [24, 26, 47].

Synovial biomarkers, including α-defensin, D-lactate, CP, ILs, synovial WBC count, and PMN%, generally demonstrate higher diagnostic accuracy than serum markers. Combinations of synovial parameters (e.g., WBC + PMN%, or α-defensin) often achieve AUC values exceeding 0.90, with sensitivities and specificities frequently above 90%. Synovial WBC count and CP consistently emerged as robust discriminators in chronic PJI, while synovial CRP often demonstrates high specificity. Importantly, synovial biomarkers directly reflect the biochemical and immunological environment within the joint, making them less susceptible to systemic influences and capable of detecting localized or low-grade infections.

Quality assessment using the QUADAS-C tool highlighted important methodological concerns. Over half of the comparisons showed a high risk of bias in patient selection (52%) and two-thirds in the reference standard (66%). The index test domain was frequently classified as Unclear (84%), while flow/timing presented High risk in 41% of comparisons, mainly due to differences in sample matrix/timing (pre-operative blood vs. intra-operative synovial fluid, fresh vs. frozen samples) and partial verification. Frequent use of data-driven thresholds and incomplete reporting of indeterminate results or blinding procedures suggests that pooled estimates may represent optimistic upper bounds of diagnostic performance. Consequently, the pooled sensitivity and specificity values reported in this meta-analysis likely reflect idealized rather than real-world diagnostic accuracy.

Systemic biomarkers are inherently susceptible to non-specific influences. Comorbid inflammatory conditions, prior antibiotic therapy, or unrelated infections may alter CRP, ESR, or D-dimer values, potentially generating false-positive or false-negative results. Conversely, infections caused by low-virulence organisms or indolent clinical courses may elicit only a limited systemic inflammatory response, reducing sensitivity.

From a pathophysiological perspective, the limited specificity of D-dimer is biologically plausible. D-dimer, a fibrin degradation product generated during fibrinolysis, reflects activation of the coagulation cascade rather than infection per se. Consequently, its levels may increase in a wide range of clinical conditions, including recent surgery, systemic inflammation, venous thromboembolism, malignancy, and advanced age. These non-specific triggers are common in arthroplasty patients and may lead to false-positive results. This mechanistic limitation explains why D-dimer, despite its theoretical appeal, is unlikely to outperform classical inflammatory markers such as CRP and ESR as a standalone diagnostic test for PJI. Collectively, these findings suggest that D-dimer should be regarded primarily as a complementary biomarker, whose diagnostic interpretation must account for sample type, infection timing, patient characteristics, and study context [20, 22, 25, 45, 46, 48–52].

Patient-specific factors, including sex, ethnicity, and comorbidities, further influence biomarker performance. For instance, sex-specific differences in D-dimer thresholds and inflammatory ratios (NLR, PLR, MLR, Platelet-to-Monocyte Ratio [PMR]) suggest that uniform cut-offs may be suboptimal [20, 24, 26, 47, 53, 54].

Synovial biomarkers, although generally more specific, are not free from limitations. Prior antibiotic therapy, comorbidities, intra-articular bleeding, or contamination with metallic debris may affect results. For example, Busch et al. reported lower synovial and serum PCT levels in septic cases than in aseptic ones, likely reflecting low-virulence infections with limited inflammatory response [19]. Renal impairment may also falsely elevate PCT. Similarly, autoimmune diseases such as rheumatoid arthritis may reduce the discriminative ability of synovial ILs, including IL-1β, IL-6, and IL-17, despite excellent performance against aseptic loosening [37].

Chronic, low-grade infections caused by organisms such as *Cutibacterium acnes* or coagulase-negative staphylococci often require adjusted diagnostic thresholds for both serum and synovial CRP due to weaker inflammatory responses [42]. Implant-related factors further complicate biomarker interpretation. Haertlé et al. demonstrated that in patients with rotating-hinge knee prostheses, standard WBC and PMN% thresholds were less reliable due to interference from metallic debris, which may impair leukocyte function [44]. In these complex revision settings, adjunctive tests such as LE and synovial glucose strips may provide additional diagnostic value. These findings emphasize that biomarker interpretation should account for implant type, mechanical wear, host factors, and clinical context [44].

In summary, traditional serum markers remain valuable for screening but are limited by non-specific influences and moderate discriminatory ability. Emerging serum biomarkers, including D-dimer and PCT, show inconsistent performance and require cautious interpretation. Synovial biomarkers, particularly WBC, PMN%, α-defensin, CP, and D-lactate, provide the most accurate and reliable diagnostic information for PJI, though interpretation must consider prior antibiotics, comorbidities, implant type, and the specific infection context. Collectively, these findings support an integrated, multimodal diagnostic approach that combines systemic and local biomarkers, tailored thresholds, and clinical judgment.

### Clinical implications

From a clinical perspective, these findings support a structured combinatorial approach based on test characteristics. Parallel testing (i.e., interpreting multiple tests simultaneously as positive if any is positive) may maximize sensitivity and is useful in early screening to reduce missed infections. Conversely, serial testing (i.e., requiring sequential positive results) increases specificity and is better suited for confirmatory diagnosis. A practical diagnostic framework may involve an initial highly sensitive marker (e.g., serum or synovial IL-6) to rule out infection, followed—if positive—by a highly specific synovial test (e.g., α-defensin or synovial CRP) to confirm infection. Within this pathway, synovial WBC and PMN% remain central, low-cost anchor tests that can be integrated at multiple decision points. Alternatively, a sequential diagnostic approach may be employed, typically starting with inexpensive systemic markers (CRP or ESR) and followed by targeted synovial assays in patients with persistent diagnostic uncertainty. Such stepwise algorithms can enhance diagnostic accuracy while maintaining cost-effectiveness and minimizing unnecessary invasive procedures. However, several clinically relevant modifiers—including prior or ongoing antibiotic therapy, autoimmune or inflammatory comorbidities, low-grade infections, implant-related factors, and patient-specific characteristics—are not fully captured and may significantly influence biomarker performance. These factors can lead to false-positive or false-negative results and currently fall outside standardized algorithmic approaches, underscoring the need for contextual clinical interpretation alongside test-based strategies.

### Methodological limitations

Overall, the available evidence suggests that the search for a single “magic bullet” biomarker for PJI is unrealistic. This reinforces the value of multimodal and stratified diagnostic strategies rather than reliance on a single marker and highlights that combining complementary serum parameters may improve overall diagnostic performance [24, 47, 53–55].

Several limitations reduce the certainty and generalizability of current findings. Most studies have a relatively small sample size and are conducted at single centers, while substantial methodological heterogeneity persists across cohorts. Differences in diagnostic thresholds, study design, and biomarker measurement techniques limit direct comparability. In many cases, diagnostic cut-offs were optimized post hoc using the same datasets without external validation, potentially inflating reported accuracy. Additional methodological concerns include incomplete reporting or exclusion of indeterminate or invalid results, as well as structural imbalances in comparative designs, such as differences in sampling procedures, timing of specimen collection, and handling of missing or discordant data. Reporting of laboratory platforms, assay calibration, and pre-analytical processing is often insufficient, making replication difficult and limiting the translation of proposed thresholds into routine clinical practice.

Despite these limitations, several promising developments are emerging. Current microbiological evidence suggests that PJI diagnostics are entering a transitional phase: while conventional culture remains the cornerstone of pathogen identification, an increasing number of studies are incorporating molecular and sequencing-based techniques to improve sensitivity, particularly for culture-negative or low-grade infections. The integration of biomarker assays with advanced molecular microbiology—such as combining metagenomic profiling with synovial α-defensin testing—represents a promising step toward a comprehensive, multimodal diagnostic framework [4]. Such approaches may enable simultaneous pathogen identification and host inflammatory profiling, potentially facilitating earlier, more accurate, and more individualized diagnosis of PJI.

### Diagnostic value of synovial biomarkers in different scenarios

Culture-negative PJI represents another big issue and diagnostic challenge in infection. In this context, synovial biomarkers may be particularly valuable, as their diagnostic signal reflects host inflammatory responses rather than direct pathogen detection. For example, alpha-defensin and calprotectin demonstrated consistently high sensitivity and specificity even in culture-negative cohorts, suggesting that these markers capture infection-related inflammatory responses not detected by conventional microbiological methods. These findings support the integration of synovial biomarkers into diagnostic algorithms when microbiological confirmation is absent or inconclusive. Beyond the issue of culture-negative infections, the diagnostic performance and optimal thresholds of synovial and serum biomarkers in PJI are significantly influenced by surgical site (hip vs. knee) and infection phase (acute/post-operative vs. chronic), precluding a one-size-fits-all approach. Acute hip PJI, particularly in the early post-operative setting, requires higher synovial WBC cutoffs (> 10,000/μL, PMN% > 90%) to optimize sensitivity and specificity amid post-operative inflammation, while chronic infections—such as knee PJI—typically use lower thresholds (> 1,200/μL, PMN% > 50–60%) and may benefit from alternative biomarkers offering superior rule-out accuracy.

For instance, in chronic knee PJI, synovial calprotectin demonstrates excellent negative predictive value (> 95–100% at 50 mg/L), outperforming WBC/PMN% for excluding infection, especially in culture-negative cases. Thus, clinicians should interpret results within the specific context—joint involved, symptom timing (acute vs. chronic), and culture-negative risk—integrating these elements for more precise, timely decision-making.

### Future research directions

Future research should focus on validating multimodal diagnostic frameworks that integrate biomarker profiles with imaging and molecular microbiology within unified clinical algorithms. Artificial intelligence (AI) approaches may further enhance diagnostic performance by identifying complex patterns that single biomarkers cannot capture, potentially enabling patient stratification according to infection probability and pathogen characteristics.

Large multicenter studies will be essential to harmonize laboratory methods, define broadly applicable diagnostic cut-offs, and evaluate the cost-effectiveness of these strategies in real-world clinical settings. Ultimately, standardized protocols combined with advanced bioinformatics tools could represent a critical step toward precision diagnostics in PJI management.

## Conclusions

The diagnosis of PJI remains a complex clinical challenge requiring the integration of multiple diagnostic modalities. Traditional serum biomarkers, such as CRP and ESR, continue to serve as practical and cost-effective first-line screening tools. In contrast, synovial biomarkers—including α-defensin, D-lactate, calprotectin, and leukocyte-based indices (WBC and PMN%)—generally demonstrate superior diagnostic accuracy and can substantially enhance diagnostic performance, particularly in culture-negative infections. However, substantial heterogeneity and risk of bias identified by QUADAS-C—particularly in patient selection, reference standards, and threshold derivation—limit the certainty and generalizability of pooled estimates. Accordingly, the reported diagnostic accuracies should be interpreted with caution. The diagnostic paradigm is progressively shifting toward multi-parameter, probabilistic frameworks that integrate clinical, microbiological, biochemical, and molecular data. In this context, the concept of a single diagnostic “gold standard” is increasingly being replaced by composite diagnostic criteria incorporating multiple sources of evidence. Future research should prioritize the development and external validation of multimodal diagnostic algorithms, potentially supported by artificial intelligence, to improve standardization and diagnostic precision in PJI. The findings of this review contribute to this evolving framework by identifying biomarkers with the strongest current evidence base and highlighting their potential integration into future composite diagnostic strategies. These results support the advancement of precision diagnostics that combine systemic and synovial biomarkers, contextualized by patient characteristics, implant type, and clinical scenario.

## Supplementary Information

Supplementary Material 1: Table S1. Serum markers and demographic characteristics. Table S2. Synovial markers and demographic characteristics. Table S3. Diagnostic performance of serum and synovial biomarkers: AUC (95% CI), cut-off values, sensitivity, and specificity by study, with reference standards and notes. Table S4. Study-level 2 × 2 tables used in the quantitative synthesis, indicating whether counts were directly derived from reported subgroup frequencies or reconstructed from published diagnostic accuracy estimates.

## Data Availability

The datasets used and/or analysed during the current study are available from the corresponding author on reasonable request.

## Abbreviations

PJI: Periprosthetic joint infection
AUC: Area Under Curve
CP: Calprotectin
WBC: White blood cell count
PMN%: Polymorphonuclear percentage
MSIS: Musculoskeletal Infection Society
ICM: International Consensus Meeting
CRP: C-reactive protein
LE: Leukocyte esterase
EBJIS: European Bone and Joint Infection Society
QUADAS-C: Quality Assessment of Diagnostic Accuracy Studies–Comparative
PRISMA: Preferred Reporting Items for Systematic Reviews and Meta-Analyses
PRISMA-DTA: Preferred Reporting Items for Systematic Reviews and Meta-Analyses of Diagnostic Test Accuracy Studies
STARD: Standards for Reporting of Diagnostic Accuracy Studies
MeSH: Medical Subject Headings
CI: Confidence intervals
TP: True positive
FN: False negative
FP: False positive
TN: True negative
LRs: Likelihood ratios
IDSA: Infectious Diseases Society of America
ESR: Erythrocyte sedimentation rate
IL: Interleukin
PCT: Procalcitonin
FDP: Fibrin degradation products
PC: Platelet count
PVR: Platelet volume ratio
NLR: Neutrophil-to-lymphocyte ratio
PLR: Platelet-to-lymphocyte ratio
MLR: Monocyte-to-lymphocyte ratio
CHR: CRP/hemoglobin ratio
ELISA: Enzyme-Linked Immunosorbent Assay
NGAL: Neutrophil Gelatinase Associated Lipocalin
APMN: Absolute Polymorphonuclear
THA: Total Hip Arthroplasty
CAR: C-reactive protein to Albumin Ratio
CHAR: C-reactive protein to Hemoglobin-Albumin Ratio
PMR: Platelet-to-Monocyte Ratio
AI: Artificial intelligence
