# Supplementary material for: Diagnostic accuracy of serum and synovial biomarker thresholds for diagnosing periprosthetic joint infection: a QUADAS-C-guided systematic review and meta-analysis
Source: Arthroplasty. 2026 May 11;8:36. doi: 10.1186/s42836-026-00396-5 (PMC13159356; doi:10.1186/s42836-026-00396-5)
Supplement: Supplementary file 1 — Supplementary Material 1: Table S1. Serum markers and demographic characteristics. Table S2. Synovial markers and demographic characteristics. Table S3. Diagnostic performance of serum and synovial biomarkers: AUC (95% CI), cut-off values, sensitivity, and specificity by study, with reference standards and notes. Table S4. Study-level 2 × 2 tables used in the quantitative synthesis, indicating whether counts were directly derived from reported subgroup frequencies or reconstructed from published diagnostic accuracy estimates. [file 42836_2026_396_MOESM1_ESM.docx]

**Table S1.** Serum markers and demographic characteristics.

| **Reference** | **Study type** | **Country/Setting & Period** | **Inclusion/Exclusion criteria** | **Patient (N enrolled/**  **N analyzed)** | **Age, years, Mean (SD)/ Mean (range)/ Mean [95%CI], Sex%F** | **Joint site (Hip/ Knee, N)** | **Infected Joints (Hip/ Knee, N)** | **Index test details (assay/brand, sample handling, blinding, timing)** | **Comparators** | **Flow & timing (verification, exclusions)** | **Microbiology** |
| --- | --- | --- | --- | --- | --- | --- | --- | --- | --- | --- | --- |
| Aimaiti et al. 2025 [21] | Retrospective single-center diagnostic accuracy | China;  tertiary referral center), Urumqi;  Jan 2010–Dec 2022 | Included: hip/knee revisions with standard pre-op work-up; PJI classified per MSIS.  Excluded: megaprosthesis; native-joint septic arthritis; acute hematogenous PJI (symptoms <3 w and >3 mo from index surgery); periprosthetic fracture/dislocation; missing data or missing serology | 841 analyzed,  PJI 435/841 (51.7%) | PJI: median 62 y (18–89), 50.3% female,  Non-PJI: median 58 y (21–87), 58.9% female | Hip (542), Knee (299) | PJI: Hip (215), Knee (220) | Serum CRP/hemoglobin ratio (CHR).  Other evaluated ratios: CRP, ESR, CRP+ESR (CE), CRP/Alb (CAR), CRP/(Hb+Alb) (C/HAR), ESR/Alb (EAR), ESR/Hb (EHR), ESR/(Hb+Alb) (E/HAR), Hb+Alb (HA).  Timing: pre-op. Assay platforms/handling/blinding: NR | CRP;  ESR;  CE;  CAR;  C/HAR;  EAR;  EHR;  E/HAR;  HA | Verification against MSIS (1 major criterion—two concordant positive cultures or sinus tract—or ≥4 minor). All had serum tests; synovial α‑defensin/synovial CRP/LE not routine | Organisms in PJI: Gram+ 66.4% (MRSA 15.2%, Staphylococcus epidermidis 14.1%, MRSE 13.8%, S. hominis 4.3%, other CoNS 3.74%); Gram− 22.4% (e.g., E. coli 6.3%, streptococci 5.5%, Enterobacter cloacae 2.9%, Klebsiella 2.9%, P. aeruginosa 1.7%); polymicrobial 6.6%; fungi 4.6%. Subgroups: CHR sensitivity 0.833 and specificity 0.830 in low-virulence infections; reduced sensitivity in diabetes and hypertension |
| Busch et al. 2020 [19] | Prospective diagnostic accuracy | Germany;  university hospital; period NR | Painful arthroplasty; low-grade focus; included adequate SF and full ICM 2018 data; excluded early post-op (<8 w), metallosis, inflammatory comorbidities (RA, HIV), prior antibiotics, insufficient fluid | 78 screened/  70 analyzed,  Aseptic joint effusion (47),  PJI (28) | Aseptic 66 ± 12.5, PJI 72 ± 11.3  Aseptic 57%,  PJI 4% | Hip, Knee, Shoulder; Aseptic  Hip 27/  Knee 18,  PJI  Hip 17/  Knee 3 | PJI according to the definition of Parvizi et al.  Hip (17), knee (3) | Serum PCT;  Synovial PCT (ELISA);  Serum CRP;  Synovial CRP (immunoturbidimetry);  Synovial α-defensin (flow cytometry);  pre-op; blinding NR | Serum CRP; synovial CRP; α-defensin | Same pre-op window; ICM 2018 reference; standardized pre-op sampling | 16 (70%) Staphylococci were found in 11 (69%), Propioni bacteria and Enterococci in each 2 (13%) and Serratia marcescens were found in 1 (6%). In 7 pz (29%) in the infection group with positive histologic specimens for infection, no bacteria could be isolated after 14 days incubation |
| Chen et al. 2022 [22] | Retrospective diagnostic accuracy | China;  single tertiary centre;  2014–2020 | Consecutive THA/TKA revisions; excluded <18 y, autoimmune disease, COPD, CAD, malignancy, anticoagulants/coagulation disorders, other infections, recent operation/trauma | 186/186 | PJI: 63 (53–71),  non-PJI: 65 (59–71)  PJI: 56%,  non-PJI: 46% | Hip (50),  knee (136) | Hip (72), knee (33)  21% (22/105) were delayed-onset PJIs (occurring after 3 mo but before 2 y post-op), and 61% (64/105) were late-onset PJIs (occurring >2 y post-op) | FDP; D-dimer; platelet count; PVR; CRP; ESR; plasma/blood assays; thresholds from ROC; platform details NR | CRP; ESR; other listed coagulation markers | MSIS 2011 reference; pre-op blood within routine window | PJI pz were further divided into culture positive and negative PJI groups. 78 were culture-positive, 27 were culture-negative |
| Fernández‑Sampedro et al. 2022 [20] | Prospective observational | Spain;  tertiary hospital; period NR | Adults undergoing revision (hip/knee); consecutive series; excluded conditions raising D‑dimer (recent VTE/PE, fracture/dislocation ≤2 w, active malignancy, other infections, ulcers, etc.) | 187 enrolled/180 analyzed, 39/180 PJI (22%)  PJI: 46%,  AL: 57% | PJI: 66.54 ± 14.13,  AL: 69.23 ± 11.14 | PJI:  Hip (27),  knee (12)  AL:  Hip (91),  knee (50) | 39 (7 early, 9 delayed, 23 late PJI) | Plasma D‑dimer (Tina‑quant, Roche Cobas c501); CRP; ESR; pre-op bloods within 2 w; thresholds from ROC | CRP; ESR | IDSA 2013 reference; pre-op within routine window | 43 microorganisms are isolated from 39 PJI pz. Only 1 pz was culture-negative and a respiratory source of infection was identified. Serological markers (ESR, CRP, D-dimer) based on the infecting organisms isolated were then evaluated |
| Hughes et al. 2022 [23] | Retrospective case series | USA;  single centre; period NR | Salvage/conversion THA ≥3 months after ORIF/prox femur or acetabulum; WBC/ESR/CRP pre-op; excluded systemic inflammatory disease, active infection/cancer, <1 y follow-up | 399 screened/  85 analyzed | NR  61% | Hip only (85) | 13 pz (13/85, 15%) were diagnosed with infection | Serum CRP; ESR; WBC; aspiration if indicated; cut-offs via ROC; blinding NR | CRP vs ESR vs WBC | Reference: pre/intra-op culture positivity or purulence; same pre-op window | Pre-op, 20% (2/10 pz) had positive cultures and 22% (2/11 pz) had positive synovial analysis. There were 19 pz with intra-op cultures drawn, and 58% (11/19 pz) were culture negative. However, 27% (3/11 pz) were culture negative intra-op but had pre-op cultures positive for infection. One patient had negative intra-op cultures and developed an infection post-op |
| Shi et al. 2022 [24] | Retrospective diagnostic accuracy; gender-stratified | China;  single centre;  period NR | Revision arthroplasty; PJI per ICM 2018 vs aseptic; excluded periprosthetic fracture/dislocation, autoimmune disease, malignancy, hematologic disease, anticoagulants, incomplete data | 271 screened/  216 analyzed [PJI (80), no-PJI (136)] | F: 65.50 (10.19),  M: 64.21 (9.62)  51% | Hip (147), knee (70) | Hip (38),  knee (42) | Serum CRP; ESR; fibrinogen; D-dimer; CRP/albumin; fasting labs; thresholds via Youden; blinding NR | Gender-stratified comparisons across listed serum markers | ICM 2018 reference; same sampling episode | Joint fluid/pus and periprosthetic tissues or bone were collected intra-op and then sent to the laboratory for aerobic and anaerobic cultures and histopathologic examination for pz with confirmed or suspected PJI |
| Wixted et al. 2023 [25] | Retrospective cohort | USA;  tertiary referral centre;  period NR | ICM 2018 definite PJI undergoing revision; control = primary TJA with D-dimer ordered and negative for DVT/PE; excluded inflammatory arthropathies; controls with DVT/PE, septic shock, pneumonia | 961 screened, 604 PJI, 135 controls reported, analyzed for sensitivities | NR | Hip and Knee, N NR | Acute PJI (50), chronic PJI (31) | D-dimer; CRP; ESR; lab timing within 90 days; blinding NR | CRP; ESR | Sensitivities in PJI cohort; non-concurrent control cohort for distributions; ICM 2018 reference | 71/81 pz (87.7%) had positive cultures. Most common isolates were MSSA (23.5%), polymicrobial infections (18.5%), MRSA (13.6%), and CoNS (9.9%) |
| Xu et al. 2022 [26] | Retrospective consecutive cohort | China;  single tertiary centre;  Jan 2008–Sep 2020 | All hip/knee revisions; exclude periprosthetic fracture, dislocation, reimplantation | 743 screened/  543 analyzed | 62.7 (12.1) | Hip and Knee, N NR | 245 | Serum CRP; ESR; IL-6; plasma fibrinogen; PC; MLR; NLR; PLR; pairwise combinations; same draw/lab; thresholds by ROC/Youden | Comparisons across listed blood markers | ICM 2013 reference; same draw for serum markers | Aerobic and anaerobic cultures with blood culture bottles. SF: S. epidermidis and S. aureus were more than 50% |

**Abbreviations:** N=number; F=female; CI=confidence interval; SD=standard deviation; NR=not reported; SF=synovial fluid; ICM=International Consensus Meeting; post-op=post-operative; w=weeks; PJI=periprosthetic joint infection; ELISA=Enzyme-Linked ImmunoSorbent Assay; PCT=procalcitonin; CRP=C-reactive protein; pre-op=pre-operative; pz=patients; THA=total hip arthroplasty; TKA=total knee arthroplasty; y=years; COPD=Chronic obstructive pulmonary disease; mo=months; FDP=Fibrin degradation product; PVR=platelet volume ratio; ESR=erythrocyte sedimentation rate; ROC=Receiver Operating Characteristic; MSIS=Musculoskeletal Infection Society; PE=pulmonary embolus; AL=aseptic loosening; IDSA=Infectious Diseases Society of America; ORIF=open reduction internal fixation; WBC=white blood cell count; vs=versus; intra-op=intraoperative; M=male; TJA=total joint arthroplasty; DVT=deep venous thrombosis; MSSA=methicillin-sensitive Staphylococcus aureus; MRSA=methicillin-resistant Staphylococcus aureus; CoNS=coagulase-negative Staphylococcus aureus; IL=interleukin; PC=platelet count; MLR=Monocyte/lymphocyte ratio; NLR=neutrophil/lymphocyte ratio; PLR=platelet count/lymphocyte ratio; VTE=Venus Thromboembolism; RA=Rheumatoid Arthritis; CAD=Coronary Artery Disease.

**Table S2.** Synovial markers and demographic characteristics.

| **Reference** | **Study type** | **Country/Setting & Period** | **Inclusion/Exclusion**  **criteria** | **Patient**  **(N enrolled/**  **N analyzed)** | **Age, years**  **Mean (SD)/**  **Mean (range)/ Mean [95%CI], Sex%F** | **Joint site (Hip/ Knee, N)** | **Infected**  **Joints (Hip/ Knee, N)** | **Index test details (assay/brand, sample handling, blinding, timing)** | **Comparators** | **Flow & timing (verification, exclusions)** | **Microbiology** |
| --- | --- | --- | --- | --- | --- | --- | --- | --- | --- | --- | --- |
| Ackmann  et al. 2022 [43] | Prospective diagnostic accuracy | Germany; tertiary revision center; period NR | Suspected chronic PJI or aseptic failure (THA/TKA) per MSIS 2018; excluded surgery <4 w, chronic systemic inflammatory disease, malignancy, other active infections, inadequate SF | 81 screened/  81 analyzed,  PJI (26), aseptic failure (55) | PJI: 75.5 (62.8–82.0), Aseptic failure: 68.0 (59.0–73.0)  49%  PJI: 40%, Aseptic failure: 60% | Hip (18), knee (63) | Knee (16), hip (10) | Serum CP; serum CRP;  serum IL-6; synovial WBC/PMN;  blood same day; thresholds for CP by ROC/Youden; blinding NR | CRP;  IL-6;  synovial WBC/PMN | Same-day bloods; synovial/intra-op samples per protocol; MSIS 2018 reference | Intra-op microbiology cultures yielded Enterobacter cloacae complex (3.8%), Corynebacterium (3.8%), S. lugdunensis (3.8%), S. epidermidis (19.2%), E. coli (3.8%), Streptococcus agalactiae (3.8%), Enterococcus faecalis (7.7%), S. caprae (3.8%), S. aureus (3.8%), S. capitis (7.7%), Streptococcus dysgalactiae (3.8%). 6 of the infected cases (23.1%) were culture-negative and 3 presented with a polymicrobial infection (11.5%) |
| Baek et al. 2023 [33] | Prospective cohort | Korea; single tertiary hospital; period NR | Suspected knee PJI vs non-PJI (pre-reimplantation and aseptic loosening); 1 excluded for insufficient sample | 38 screened/  37 analyzed | Md=76 (62-83), PJI: 73 [70–79], non-PJI: 78 [71–81]  81% | Knee only (37) | 18 | α-defensin (ELISA, frozen–thawed aliquots); LE strip; synovial WBC/PMN on fresh samples; serum ESR/CRP; 16S rRNA MTP; blinding unclear | ESR/CRP; synovial WBC; PMN%; LE; cultures; MTP | ICM 2013 reference; same encounter; processing differed across tests (fresh vs frozen) | Among the 18 PJI episodes, 13 were culture-positive cases (11, synovial culture only; 1, blood culture only; 1, both samples); the pathogens identified through culture were also detected via MTP in 8 cases. Identified with MTP: E. coli (10), S. aures (8), E. faecalis (1), Bacteroides spp (2) |
| Baker et al. 2022 [29] | Retrospective analysis of prospectively collected cohort | USA; single high-volume center; period NR | Candidates for revision with pre-op aspiration; PJI by ICM 2018; excluded <90 days, acute hematogenous, incomplete data, no aspiration | 621 reviewed/588 analyzed, PJI (194), aseptic failure (394) | 67 (37 to 91)  44.5% | Hip (68), knee (520) | Hip (32), knee (162) | Synovial CRP (ELISA; cut-off 6.9 mg/L); Serum CRP; ESR; PMN%; WBC; α-defensin; blinding NR | Head-to-head among listed markers | Pre-op window; ICM 2018 reference; predefined ICM cut-offs | NR |
| Felstead et al. 2022 [39] | Prospective diagnostic accuracy (sample-level) | UK; 3 secondary care hospitals; period NR | Included synovial aspirates with accompanying PPT; excluded aspirates without PPT and incomplete data | 134 patients (161 samples)/161 | NR | Hip (49), knee (112) | Hip: Chronic (6), Early post-op infection (3), Acute haematogenous infection (9). Knee: Chronic (4), Early post-op infection (8), Acute haematogenous infection (21) | Synovial CRP (Afinion; thresholds via ROC/Youden post hoc); Synovial WCC (IRIS IQ; 3,000×10^6/L; 4,500×10^6/L for THR sensitivity); same synovial sample; pre/intra-op; blinding not stated | Dual rule in parallel (CRP>10 mg/L OR WCC>3,000×10^6/L) | Reference: MSIS major criteria; same-sample head-to-head; pre-op aspiration or intra-op fluid; thresholds derived post hoc | NR |
| Fuchs et al. 2022 [27] | Prospective diagnostic accuracy (preliminary single-centre) | Germany; university hospital; period NR | Adults with painful THA/TKA or scheduled revision; excluded specimen clotting, insufficient synovial volume, refusal | PJI: 70 (54-86), Aseptic: 72 (40-90) | PJI: 70 (54-86), Aseptic: 72 (40-90)  PJI: 84,72%, aseptic: 61% | Hip and Knee, N NR  PJI: Hip (6), knee (12)  Aseptic:  Hip (11), knee (43) | PJI: 18 (25%); between them: 9 delayed (3–24 mo) and 4 late (>24 mo)   Aseptic: 54 (75%) | Synovial D-lactate (colorimetric, 450 nm); synovial leukocyte count and granulocyte %; same aspirate split; thresholds via ROC/Youden; blinding NR | Synovial leukocyte count; % granulocytes; cultures; histopathology | Institutional composite (clinical, synovial WBC/PMN, histopathology, cultures incl. sonication); same episode | 3–5 periprosthetic tissue biopsies collected intra-op for microbiological and histopathological analysis. Coagulase-negative staphylococci (4); S. aureus (1); Streptococcus spp. (4); Enterococcus spp. (1); Enterobacteriaceae (4); Pseudomonas aeruginosa (1); Other – Culture-negative (5); Polymicrobial infection (2) |
| Grzelecki et al. 2023 [41] | Prospective | Poland; single academic centre; period NR | Revision THA/TKA suspected PJI or aseptic; excluded PPF/dislocation; second-stage PJI; inconclusive PJI; early or hematogenous <4 w; chronic inflammatory disease; active malignancy; systemic infection; refusal/missing data | 157 enrolled/  110 analyzed, 42/110 PJI (38%) | 75  PJI: 69 (64-74)  66%  arTJA: 79 (54), PJI: 45 (19) | Knee, % (n) arTJA, 79 (54)  PJI, 64 (27)  Hip (29), knee (81) | PJI, 64 (27)  PJI group: Hip (15), knee (27) | D‑lactic acid strip (QuantiQuick, BioAssay Systems); LE strip 10 EA (ARKRAY); LE strip BM 10 (BioMaxima); same sample; manual visual reading by 3 assessors; not fully blinded; some samples centrifuged; D‑lactate thresholds via Youden | LE strips (2 brands) | ICM 2018 reference (LE not used as minor criterion); pre/intra-op sampling | MSSA, cultured in 24% (10/42);  Hip: 20(3), knee: 26(7). Pathogen: Hip (15), knee ( 27); MSSA: 20 (3), 26 (7); MRCNS: 20 (3), 15 (4); MSCNS: 13 (2), 7 (2); S. agalactiae: 7 (1), 7 (2); MSSE: 7 (1), 4 (1); E. faecalis: 7 (1), 4 (1); MRSA: 7 (1), 0;  K. pneumonia: 0, 4 (1); E. faecium: 0, 4 (1);  E. cloacae: 0, 4 (1); P. oralis: 7 (1), 0;  C. acnes: 7 (1), 0; S. mitis: 0, 4 (1); Streptococcus G: 7 (1), 0; S. haemoliticus: 0, 4 (1) |
| Güneş et al. 2025 [28] | Prospective diagnostic accuracy | Turkey; multicenter (state/university hospitals); Sep 2018–Jun 2021 | Included: chronic knee PJI per 2018 ICM; ≥2 mL synovial fluid obtainable. Controls: primary knee OA undergoing primary TKA. Excluded: coagulation disorders, prosthetic heart valves, recent skin ulcers/hemorrhage/ecchymosis/trauma, comorbid inflammatory diseases, active cancer; samples with protocol deviations discarded | 69 analyzed, PJI 17/69 (24.6%) | All: mean 68.1 y (50–84), 24.6% female,  PJI: mean 68.8 y (52–82), 47.1% female,  Controls: mean 68.4 y (50–84), 17.3% female | Knee only: 69 total (PJI 17, Controls 52) | Chronic  Knee (17) | Synovial fluid D-dimer by ELFA (VIDAS D-dimer Exclusion II, bioMérieux). Handling: intraoperative aspiration before arthrotomy; ≥2 mL; EDTA tube for cytology and 3.8% sodium-citrate tube for D-dimer; centrifuge 1000 g 10 min; supernatant stored −80°C; assay range 0–1,500,000 ng/mL; dilute 1:4 if >upper limit; dilution factor applied. Timing: synovial intraoperative; blood preoperative. Blinding: NR | Serum CRP; ESR;  plasma D-dimer | ICM 2018 reference; pre-/peri-op sampling; same episode | Microbiology collected but not reported in detail (NR) |
| Haertlé et al. 2022 [44] | Prospective diagnostic study | Germany; single university hospital; 2014–2017 | Post-arthroplasty effusion; included adults with prosthesis and effusion; excluded traumatic effusion, insufficient volume, analysis >6 h post-aspiration | 145/145 | 67.0 ± 15.7  51% | Hip (44), knee (98) | Hip (19), knee (26) | LE strip (++/+++ positive); glucose strip (negative); same strip platform; centrifuged bloody samples; thresholds pre-specified; blinding NR | Combined rule (LE++/+++ AND glucose negative) | Modified MSIS (no intra-op criteria); ED/outpatient mix; same visit | 42/46 SF classified as septic showed microbial growth in culture. The most detected pathogens were Staphylococcus strains, e.g., S. aureus and S. epidermidis |
| Huang et al. 2022 [35] | Prospective diagnostic accuracy | China; single orthopaedic infection center; period NR | Revision hip/knee for PJI or aseptic loosening; excluded incomplete data, SF <1 mL, heavily blood-contaminated SF, recurrent infection | 83 screened/78 analyzed,  PJI (50; 26 chronic),  AF (28) | 65 (19-95)  53.9% | PJI:  Hip (24), knee (26) | Chronic: Hip (14), knee (12) | Synovial NGAL (ELISA on frozen aliquots); SF-WBC and PMN% on fresh samples; Serum CRP and ESR; thresholds via ROC/Youden; blinding NR | Serum CRP; ESR; SF-WBC; PMN% | ICM 2013 reference; pre-/peri-op sampling; same episode | A total of 0.1 ml of SF was inoculated into blood plates for bacterial and fungal culture. The remaining SF was injected into Bactec Plus/F aerobic or Bactec Peds Plus/F blood culture bottles and anaerobic blood culture bottles. The plates and bottles were incubated for 14 days in a Bactec 9050 automatic incubator. The periprosthetic tissue was cut into pieces, added to the broth for grinding, and then cultured for aerobic and anaerobic bacteria on a blood plate for 14 days. The Vitek II system was used for microbial identification and antibiotic susceptibility testing. A total of 40 cases were positively cultured. The most common pathogen was S. aureus in 10 (25%) pz |
| Lazic et al. 2022 [36] | Prospective cohort (PPF with loosening) | Germany; university hospital (TUM); period NR | THA/TKA PPF (specific classifications); 30 consecutive cases; excluded cases missing both SF and histopathology (n=2) | 30/30,  14 PJI/16 aseptic (47%) | 72.3 (14.8),  Septic cases (14): 71.8 ± 14.0, Aseptic cases (16): 72.7 ± 15.1  70% | Hip (23), knee (7) | Hip (10), knee (4) | CP LFT (Lyfstone); smartphone reader; surgeons blinded; manufacturer threshold 50 mg/L; ROC suggested 76 mg/L | None (single-test accuracy) | Modified EBJIS 2021 adapted for PPF; intraoperative sampling | 1 THA S. epidermidis, 1 THA S. saccharolyticus, 1 THA S. warneri |
| Mihalič et al. 2020 [34] | Prospective consecutive series | Slovenia; Valdoltra Orthopaedic Hospital; Mar 2012–Jan 2014 | Revision THA/TKA for any reason except periprosthetic fracture; 1 shoulder excluded | 50 joints (49 pz) enrolled/49 joints (48 pz) analyzed | 68(10)  53% | Hip (24),  knee (25) | Hip (6),  knee (5) | Synovial WBC count (manual, Neubauer chamber); Synovial %PMN; Synovial IL‑6 (CLIA, Immulite 2000); pre/intra-op aspiration; cultures 14 days; blinding NR | Synovial WBC; %PMN vs IL‑6 (head-to-head) | Reference: modified criteria (sinus tract OR histology >5 PMN in ≥10 HPF OR same organism in ≥2 of ≥4 samples); same aspirate for tests | 4 tissue samples were collected during surgery and cultured for 14 days on solid and liquid media |
| Qin et al. 2022 [37] | Prospective diagnostic accuracy | China; single academic center; period NR | Aseptic loosening revisions; PJI; active RA after arthroplasty (DAS28>3.2); adequate SF ≥1 mL; excluded trauma/dislocation, ulcers, coagulation issues | 102/102    102:  39 underwent aseptic revision (group A),  37 diagnosed with PJI and underwent stage I prosthesis removal and bone cement filling (group B),  26 diagnosed with active RA (group C) | Group A: 62.0 ± 8.386,  group B: 55.8 ± 4.763,  group C: 64.9 ± 6.817  Group A: 56%,  group B: 88.5%,  group C: 57% | Hip: 23 (group A), NA (group B), 16 (group C)  Knee: 16 (group A), 26 (group B), 21 (group C) | Hip (16),  knee (21) | Synovial IL-1β, IL-2, IL-4, IL-6, IL-8, IL-10, IL-12, IL-17 (ELISA on same sample); blinding NR | Panel comparisons across ILs | MSIS (2011/ICM-based) reference; same sampling episode | NR |
| Pascal et al.  2025 [40] | Prospective comparative diagnostic accuracy study | France;  single-center tertiary referral | Adults with arthroplasty and suspected OAI with “complex microbiological diagnosis” (discordant punctures, prior sterile puncture with high suspicion, or ongoing antibiotics); TAD required for inclusion. Excluded protocol deviations, missing data, suspected metallosis | 79/79 | Mean 66±13.5 y,  range 26–89  34% | Hip (41), Knee (36) | Hip (14),  Knee (11) | α‑defensin lateral flow (Synovasure, Zimmer Biomet) and leukocyte esterase strip (Multistix 8SG, Siemens).  Fresh SF from preo-p aspiration; TAD always, TLE only if fluid clear; no centrifugation.  Readout: TAD 10 min (two lines=positive); TLE 2 min (≥++=positive).  Performed and read immediately by surgeon; blinding NR | Reference standard: MSIS 2018 PJI criteria.  Head‑to‑head agreement TAD vs TLE (Cohen’s κ) | Prospective single‑center (CRIOAC Lille), Mar 2018–Dec 2023; adults with suspected OAI in “complex microbiology” (discordant taps, culture‑negative despite suspicion, on antibiotics).  Same‑session aspiration + rapid tests; three aliquots sent for culture; diagnosis validated in multidisciplinary meeting.  Exclusions: protocol deviation, missing data, suspected metallosis.  TLE not feasible in ~30% due to hemarthrosis | Major organisms in infected: S. epidermidis (9), S. haemolyticus (3), Cutibacterium acnes (3), S. capitis (2), S. caprae (2), Citrobacter freundii (2), plus single cases (e.g., S. aureus, E. coli, Enterobacter cloacae, streptococci, Peptoniphilus, Corynebacterium striatum) |
| Sebastian et al. 1  2025 [30] | Retrospective (analysis of prospectively maintained registry/database) | Austria;  single-center tertiary | Included: rTHA/rTKA with preop synovial aspiration and synovial CRP plus complete ICM 2018 data (clinical, labs: serum CRP, synovial CRP, synovial WBC, synovial PMN; op notes; microbiology; pathology). Excluded: reimplantations, spacer exchanges, and ICM‑inconclusive cases | 340/340 | THA 69.0 (13.8), TKA70.8 (9.69)  60.6% | Hip (83),  Knee (304) | Hip (42),  Knee (47) | Synovial CRP on fresh aspirate; synovial WBC/synovial PMN on Sysmex XN‑550; serum CRP on Abbott Architect Plus c4000; pre-op same‑encounter testing; blinding NR | Serum CRP; synovial WBC; synovial PMN% (ICM 2018 thresholds referenced) | ICM 2018 applied without serum ESR (not collected); ICM‑inconclusive excluded; other indeterminate handling NR | Culture positive in 146/189 (77%); most common S. epidermidis (n=38); high‑virulent 59/146 (40%); low‑virulent 87/146 (60%); 14‑day aero/anaerobic cultures from SF, ≥5 tissues, and sonication aliquots |
| Sebastian et al. 2  2025 [31] | Retrospective (analysis of prospectively maintained registry/database) | Austria;  single-center tertiary | Included: rTHA/rTKA with pre-op aspiration and available serum CRP, synovial WBC, PMN%, surgical details, microbiology, histology; classified by EBJIS and ICM 2018. Excluded: reimplantations and spacer exchanges; for ROC accuracy also excluded EBJIS “infection likely” (n=20) and ICM “inconclusive” (n=39), metallosis (n=10), polyethylene wear (n=12) | 616 revisions (177 hip; 439 knee). EBJIS: infected 325/616 (52.8%), unlikely 271 (44%), likely 20 (3.2%). ICM 2018: infected 308/616 (50.0%), not infected 269 (43.7%), inconclusive 39 (6.3%) | Mean age 70.1±10.9 y  59.2% | Hip (177), Knee (439) | EBJIS:  Hip (102),  Knee (223)  ICM 2018:  Hip (96),  Knee (212) | Synovial absolute PMN (APMN) = (synovial WBC × PMN%)/100. Measurement: WBC and PMN% on Sysmex XN‑550; serum CRP on Abbott Architect plus c4000; α‑defensin method not specified.  Timing: pre-op aspiration. Cut-offs and accuracy—EBJIS: Hip 783.6 cells/uL.; Knee 549 cells/uL. ICM 2018: Hip 783.6 cells/uL; Knee 594.2 cells/uL | Serum CRP; synovial WBC; synovial PMN%; synovial α‑defensin; subgroup analyses for UPIC, UNIC, high/low-virulence, acute vs chronic | Verification: EBJIS 2021 and ICM 2018 criteria. Intra-op: ≥5 periprosthetic tissue samples for culture, ≥2 for histology; aerobic/anaerobic cultures up to 14 days (0.1 mL plating); histology per Krenn & Perino. ROC analyses excluded likely/inconclusive, metallosis, PE wear | EBJIS infected n=325: culture-negative 80 (24.6%), culture-positive 245 (75.4%). Of 306 isolates: aerobic Gram+ 59.8% (S. epidermidis 66, S. aureus 38, others), Gram− 11.1% (e.g., E. coli 9, P. aeruginosa 8, Proteus 5, Enterobacter 3, etc.), anaerobes 16.6% (Cutibacterium acnes 37, C. avidum 7, etc.), fungi 4% (Candida spp). ICM results are similar (culture-negative 22.4%). High-virulence pathogens identified in ~39.5% (EBJIS) and ~41% (ICM) of septic procedures |
| Suren et al. 2023 [38] | Prospective diagnostic accuracy | Germany; national referral center; period NR | Scheduled exchange THA/TKA for chronic/low-grade PJI vs aseptic failure; included multiple prior surgeries, severe osteolysis, wear disease; excluded early post-op or acute hematogenous, surgery/dislocation <3 mo, periprosthetic fracture needing exchange, antibiotics <2 w, dry taps | 137/137 | 67 (±13) years    F (49);  M (88) | Hip (53), knee (84)  74 Primary (30 THA, 44 TKA), 63 had revision or tumour implants (22 THA, 38 TKA) | 99 (72.8%) not infected, 34 (25%) infected (16 Hip, 18 Knee), 4 (2.9%) inconclusive | CP LFT (Lyfstone); measured intra-op; surgeons blinded; manufacturer thresholds; “moderate risk” counted negative | Pre-op ICM components (CRP, ESR, SF-WBC/diff, cultures) | Reference: ICM 2018 post-op score; index intra-op; composite pre-op data for comparison | Aerobic and anaerobic cultures of SF; 5 biopsies retrieved during surgery for conventional culture; explanted components were sent for sonication and culture of the sonication fluid |
| Theil et al. 2024 [32] | Retrospective single-centre | Germany; tertiary referral; period NR | MoM RHK revisions; included pre-op aspiration and in-house cell count; excluded outside lab counts, lab failure, oncology reconstructions | 120 screened/108 analyzed | 70 (IQR 25-94)  61% | Knee only (RHK, 108) | Infected 59/108 (55%), AL 38/108 (35%),  Instability 11/108 (10%) | Synovial WBC and PMN% measured on same analyzer; thresholds evaluated vs MSIS/ICM/EBJIS; blinding NR | Internal comparison WBC vs PMN% | Same encounter; accepted MSIS 2011/2018 classification; EBJIS thresholds referenced | Intra-op microbiological culture results |
| Wang et al. 2021 [42] | Prospective diagnostic accuracy | China; single tertiary center; period NR | Revision for chronic PJI or aseptic loosening; included if met 2013 MSIS; excluded malignancy, autoimmune disease, renal failure, chronic infections (HIV/HCV), antibiotics <2 w, inadequate SF, infection ≥1 y after aseptic revision | 122 screened/97 analyzed (58 Aseptic and 39 Infected) | Aseptic: 69.37 (12.63);  PJI: 73.28 (6.18)  NR | Hip (61),  knee (36) | Hip (28),  knee (11) | Serum CRP; Synovial CRP; ESR; synovial PMN%; blood same day; knee SF same day; hip SF intra-op; thresholds by ROC/Youden; blinding NR | Head-to-head among listed markers | Same-day head-to-head; MSIS 2013 reference; same encounter across tests | The most isolated pathogens were coagulase-negative staphylococci (13, 38.24%), S. aureus (11, 32.35%), S. epidermidis (3, 8.82%), MRSA (2, 5.88%), C. tropicalis (2, 5.88%), Streptococcus gagalactiae (2, 5.88%) and Carbapene-resistant A. baumannii (1, 2.95%) |

**Abbreviations:** N=number; NR=not reported; CI=confidence interval; SD=standard deviation; PJI=periprosthetic joint infection; THA=total hip arthroplasty; TKA=total knee arthroplasty; MSIS=Musculoskeletal Infection Society; w=weeks; SF=synovial fluid; CP=Calprotectin; CRP=C-reactive protein; IL=interleukin; WBC=white blood cell count; PMNs=polymorphonuclear leukocytes; ROC=Receiver Operating Characteristic; intra-op=intraoperative; vs=versus; ELISA=Enzyme-Linked ImmunoSorbent Assay; LE=leukocyte esterase; ESR=erythrocyte sedimentation rate; MTP=metagenomic microbiome profiling; pre-op=pre-operative; ICM=International Consensus Meeting; PPT=periprosthetic tissue; THR=total hip revision; mo=months; PPF=periprosthetic fracture; MRCNS= methicillin-resistant coagulase-negative Staphylococcus; MSCNS=methicillin-sensitive coagulase-negative Staphylococcus; MSSA=methicillin-sensitive Staphylococcus aureus; MSSE=methicillin-sensitive Staphylococcus epidermidis; MRSA=methicillin-resistant Staphylococcus aureus; h=hours; mo=months; WCC=white cell count; NGAL=neutrophil gelatinase-associated lipocalin; PMN%=polymorphonuclear neutrophil percentage; peri-op=peri-operative; pz=patients; LFT=lateral flow test; EBJIS=European Bone & Joint Infection Society; RA=rheumatoid arthritis; post-op=post-operative; RHK=rotating-hinge knee.

**Table S3.** Diagnostic performance of serum and synovial biomarkers: AUC (95% CI), cut-off values, sensitivity, and specificity by study, with reference standards and notes.

| **Reference** | | **Index test** | | **AUC**  **(95% CI)** | **Cut-off**  **(units)** | **Sensitivity**  **(%; 95% CI)** | **Specificity**  **(%; 95% CI)** | **Reference standard/Notes** |
| --- | --- | --- | --- | --- | --- | --- | --- | --- |
| Ackmann et al. 2022 [43] | | CP | | 0.899 (0.830–0.968) | 9,910 ng/mL (Youden) | 81 (NR) | 91 (NR) | MSIS 2018/chronic PJI only  Immunoturbidimetry |
| Aimaiti et al. 2025 [21] | | Serum CRP | | 0.860 (0.843–0.893) | 11.050 mg/L | 78.1 (73.9–81.8) | 86.4 (82.6–89.5) | MSIS 2018  841 hip/knee revisions  Retrospective single-center  Pre-op serum sampling |
|  |  | Serum ESR | | 0.833 (0.806–0.860) | 39.500 mm/h | 68.9 (64.3–73.2) | 84.5 (80.5–87.7) |  |
|  |  | CRP + ESR  (CE, composite) | | 0.866 (0.841–0.891) | 0.475 (dimensionless) | 79.2 (74.6–82.5) | 83.7 (79.4–86.9) |  |
|  |  | CRP/Albumin ratio  (CAR) | | 0.873 (0.849–0.898) | 0.287 (dimensionless) | 79.7 (75.6–83.3) | 86.4 (82.6–89.5) |  |
|  |  | CRP/Hemoglobin ratio (CHR) | | 0.872 (0.847–0.896) | 0.078 (dimensionless) | 81.3 (77.3–84.8) | 83.9 (79.9–87.3) |  |
|  |  | CRP/(Hemoglobin + Albumin) ratio  (C/HAR) | | 0.872 (0.848–0.897) | 0.061 (dimensionless) | 81.3 (77.3–84.8) | 84.9 (81.0–88.2) |  |
|  |  | ESR/Albumin ratio  (EAR) | | 0.846 (0.820–0.873) | 0.893 (dimensionless) | 77.9 (73.6–81.6) | 80.3 (76.0–83.9) |  |
|  |  | ESR/Hemoglobin ratio (EHR) | | 0.835 (0.808–0.862) | 0.254 (dimensionless) | 78.3 (74.1–82.1) | 73.6 (69.0–77.8) |  |
|  |  | ESR/(Hemoglobin + Albumin) ratio  (E/HAR) | | 0.840 (0.813–0.867) | 0.203 (dimensionless) | 76.7 (72.4–80.6) | 77.6 (73.1–81.4) |  |
|  |  | Hemoglobin + Albumin (HA, composite sum) | | 0.715 (0.680–0.750) | 154.950 (composite units) | 49.4 (44.7–54.3) | 15.5 (12.2–19.4) |  |
| Baek et al. 2023 [33] | | CRP (serum) | | NR | 10 mg/L | 76.5 (56.3–96.6) | 77.8 (58.6–97.0) | ICM 2013  TKA only |
|  |  | ESR (serum) | | NR | 30 mm/h | 70.6 (49.0–92.2) | 77.8 (58.6–97.0) |  |
|  |  | Synovial WBC | | NR | — | 94.4 (NR) | 100.0 (NR) | Parvizi et al. 2014 |
|  |  | Synovial PMN% | | NR | — | 89.9 (NR) | 92.9 (NR) |  |
|  |  | LE (strip) | | NR | — | 66.7 (NR) | 100.0 (NR) |  |
|  |  | α‑defensin (ELISA) | | 0.93 (NR) | 1.58 mg/L | 94.4 (NR) | 89.5 (NR) |  |
|  |  | MTP | | NR | — | 100.0 (NR) | 55.6 (NR) |  |
| Baker et al. 2022 [29] | | CRP (serum) | | 0.926 (0.903–0.949) | 1.0 mg/dL | 83.5 (NR) | 88.3 (NR) | ICM 2018  Mixed cohort THA/TKA |
|  |  | Synovial CRP | | 0.951 (NR) | 6.9 mg/L | 74.2 (NR) | 98.0 (NR) |  |
|  |  | Synovial PMN% | | 0.973 (NR) | 80% | 86.4 (NR) | 99.2 (NR) |  |
|  |  | Synovial WBC | | 0.978 (NR) | 3,000 cells/μL | 91.7 (NR) | 98.7 (NR) |  |
|  |  | α-defensin (LFT) | | 0.925 (NR) | Qualitative (pos/neg) | 98.6 (NR) | 86.9 (NR) |  |
| Busch et al. 2020 [19] | | PCT (serum) | | NR | 0.1 ng/mL | 26 (NR) | 81 (NR) | ICM/MSIS 2018  Siemens immunoassay  (LOD 0.02 ng/mL) |
|  |  |  |  | NR | 0.3 ng/mL | 17 (NR) | 84 (NR) |  |
|  |  |  |  | NR | 0.5 ng/mL | 13 (NR) | 91 (NR) |  |
|  |  | CRP (serum) | | NR | 0.5 mg/dL | 57 (NR) | 81 (NR) | ICM/MSIS 2018  Immunoturbidimetry |
|  |  | α‑defensin (ELISA) | | NR | 4.8 mg/L | 52 (NR) | 88 (NR) | ICM/MSIS 2018 |
|  |  | Synovial PCT | | NR | 1.0 ng/mL | 87 (NR) | 0 (NR) |  |
|  |  | Synovial CRP | | NR | 6.9 mg/L | 26 (NR) | 100 (NR) |  |
| Chen et al. 2022 [22] | | D-dimer | | 0.571 (0.487–0.655) | 0.685 mg/L | 48.6 (NR) | 71.2 (NR) | MSIS  Compared with CRP/ESR |
|  |  | D-dimer (THA) | | 0.533 (0.435–0.632) | 0.685 mg/L | 44.4 (NR) | 71.9 (NR) | THA subgroup |
|  |  | D-dimer (TKA) | | 0.665 (0.515–0.832) | 1.55 mg/L | 27.3 (NR) | 100.0 (NR) | TKA subgroup |
|  |  | FDP (plasma) | | 0.805 (0.740–0.870) | 3.935 mg/L | 78.6 (NR) | 75.0 (NR) | MSIS  Compared with CRP/ESR |
|  |  | PLT | | 0.703 (0.627–0.780) | 216 ×10^9/L | 65.7 (NR) | 67.9 (NR) |  |
|  |  | PVR | | 0.704 (0.627–0.780) | 21.053 (unitless) | 76.0 (NR) | 59.3 (NR) |  |
|  |  | CRP | | 0.882 (0.831–0.932) | 9.55 mg/L | 91.4 (NR) | 72.5 (NR) | — |
|  |  | ESR | | 0.824 (0.765–0.883) | 67.5 mm/h | 67.6 (NR) | 83.8 (NR) | — |
| Felstead et al. 2022 [39] | | Synovial CRP | | 0.911 (NR) | 10 mg/L | 88.24 (NR) | 93.14 (NR) | MSIS 2013/second-stage samples excluded |
|  |  | Synovial WBC | | 0.960 (NR) | 3,000 cells/μL | 94.12 (NR) | 87.25 (NR) |  |
| Fernandez‑Sampedro et al. 2022 (Late ≥90 d) [20] | | D-dimer | | 0.767 (0.665–0.870) | >1167 ng/mL | 69.5 (51–88) | 75.3 (69–83) | IDSA + MSIS/ICM 2018 |
|  |  | CRP | | 0.863 (0.772–0.953) | >1 mg/dL | 82.6 (67–98) | 82.1 (76–88) |  |
|  |  | ESR | | 0.767 (0.592–0.871) | >15 mm/h | 73.9 (56–92) | 72.8 (65–80) |  |
|  |  | D-dimer + CRP | | 0.855 (0.782–0.928) | — | 65.2 (45.7–84.7) | 92.7 (88.3–97) |  |
|  |  | D-dimer + ESR | | 0.736 (0.633–0.882) | — | 65.2 (45.7–84.7) | 88.3 (82.9–93.7) |  |
|  |  | CRP + ESR | | 0.783 (0.660–0.919) | — | 73.9 (56–91.8) | 87.8 (82.4–93.3) |  |
| Fuchs et al. 2022 [27] | | D‑lactate | | 0.92 (0.86–0.99) | 3.6 mg/L (≈0.04 mmol/L) | 90.7 (79.7–96.9) | 83.3 (58.6–96.4) | Comparator: synovial leukocytes and % granulocytes |
|  |  | Synovial leukocytes + % granulocytes | | 0.90 (0.79–1.00) | — | 93.7 (69.8–99.8) | 79.6 (64.0–89.4) |  |
| Grzelecki et al. 2023 [41] | | LE (10EA strip) | | NR | Pos per strip thresholds | 81 (66–91) | 99 (91–100) | ICM 2018 (+) =≥ 75  (++) =≥ 250  (+++) =≥ 500 leukocytes/μL |
|  |  | LE (BM10 strip) | | NR | Pos per strip thresholds | 81 (65–91) | 97 (89–100) | ICM 2018 (+) =≥ 70  (++) =≥ 125  (+++) =≥ 500 leukocytes/μL |
|  |  | D‑lactate (rapid test) | | NR | Categories ≥22.5/≥45/≥90/≥180 mg/L | NR | NR | ICM 2018 Distribution by category reported  No aggregate Se/Sp |
| Güneş et al. 2025 [28] | | Synovial D-dimer | | 0.992 (0.992–1.000) | 236,804 ng/mL | 100 (93.2–100) | 94.12 (71.3–99.9) | ICM 2018  Verification with ≥5 intra-op periprosthetic tissue cultures incubated up to 14 days  Synovial sample  DeLong: superior to comparator serum CRP (P=0.015) and plasma D-dimer (P=0.031), not significantly different from ESR (P=0.059) |
| Haertlé et al. 2022 [44] | | LE (strip) | | 0.792 (NR) | Neg if “−/+” | 67.4 (NR) | 90.1 (NR) | Modified MSIS (intra‑op excluded)/Same LE + Glucose: AUC 0.740; Se 50; Sp 97.9 |
|  |  | Synovial glucose | | 0.723 (NR) | Semiquantitative (mmol/L scale) | 56.5 (NR) | 87.9 (NR) |  |
| Huang et al. 2022 [35] | | CRP (serum) | | 0.89 (0.82–0.96) | 8.7 mg/L | 79.6 (61.1–86.7) | 89.3 (71.8–97.7) | ICM 2013  THA+TKA |
|  |  | ESR (serum) | | 0.90 (0.84–0.97) | 35.5 mm/h | 83.7 (72.8–94.1) | 85.5 (NR) |  |
| Huang et al. 2022 [35] | | Synovial NGAL | | 0.98 (NR) | 263.5 ng/mL | 92.9 (NR) | 98.0 (NR) | ICM 2013 |
|  |  | Synovial WBC | | 0.99 (NR) | 3,005 cells/μL | 89.8 (NR) | 100.0 (NR) |  |
|  |  | Synovial PMN% | | 0.95 (NR) | 57.60% | 93.9 (NR) | 89.3 (NR) |  |
| Hughes et al. 2021 [23] | | CRP (serum) | | 0.840 (NR) | 7.1 mg/L | 80.0 (44.4–97.5) | 88.0 (78.4–94.4) | Pre‑op CRP  Other cut-offs 9.4 and 14.2 mg/L |
| Lazic et al. 2022 [36] | | CP (LFT) | | 0.69 (NR) | 76 mg/L (Youden); kit threshold 50 mg/L | 71 (48–95) | 81 (54–96) | EBJIS 2021 modified |
| Mihalič et al. 2020 [34] | | Synovial IL‑6 | | 0.861 (NR) | 2,300 pg/mL | 73 (NR) | 95 (NR) | Cut-offs derived from Trampuz et al. |
|  |  | Synovial PMN% | | 0.944 (NR) | ≥65% | 82 (NR) | 97 (NR) |  |
|  |  | Synovial WBC | | 0.944 (NR) | 1,700 cells/μL | 82 (NR) | 97 (NR) |  |
| Pascal et al. 2025 [40] | | α‑defensin (lateral flow, Synovasure) — all participants | | NR | Qualitative (positive = two bands) | 89 (NR) | 89 (NR) | MSIS 2018  Immediate test on fresh aspirate |
|  |  | α‑defensin (lateral flow) — clear‑fluid subgroup | | NR | Qualitative | 87 (NR) | 88 (NR) | MSIS 2018  Subset where TLE feasible |
|  |  | LE (urine strip) — clear‑fluid subgroup | | NR | ≥2+ (colorimetric scale) | 87 (NR) | 81 (NR) | MSIS 2018  Performed only if no hemarthrosis |
| Qin et al. 2022 [37] | | Synovial IL‑1β | | 0.959 (0.918–0.999) | 71.03 pg/mL | 95.6 (83.0–99.4) | 86.2 (69.4–94.0) | MSIS  Groups: PJI, aseptic, active RA |
|  |  | Synovial IL‑2 | | 0.880 (0.795–0.966) | 6.50 pg/mL | 81.1 (65.0–90.5) | 85.7 (68.5–94.0) |  |
|  |  | Synovial IL‑6 | | 0.951 (0.901–1.000) | 1,327 pg/mL | 90.0 (74.4–96.5) | 89.3 (72.8–96.3) |  |
|  |  | Synovial IL‑8 | | 0.962 (0.917–1.000) | 1,033 pg/mL | 86.1 (71.3–93.9) | 100.0 (88.7–100.0) |  |
|  |  | Synovial IL‑10 | | 0.851 (0.755–0.946) | 1.48 pg/mL | 86.5 (72.0–94.1) | 74.1 (55.3–86.8) |  |
|  |  | Synovial IL‑17 | | 0.736 (0.612–0.861) | 2.95 pg/mL | 60.0 (43.6–74. 5) | 88.5 (71.0–96.0) |  |
| Sebastian et al. 1 2025 [30] | Hip  chronic >90 d | Synovial CRP | | 0.90 (NR) | 3.00 mg/L | 85.0 (NR) | 93.0 (NR) | ICM 2018  ROC‑derived threshold |
|  |  | Synovial WBC | | 0.87 (NR) | 1,730 cells/mL | 81.0 (NR) | 93.0 (NR) |  |
|  |  | Synovial PMN% | | 0.87 (NR) | 60.05% | 81.0 (NR) | 90.0 (NR) |  |
|  | Knee  chronic >90 d | Synovial CRP | | 0.88 (NR) | 1.65 mg/L | 81.0 (NR) | 90.0 (NR) |  |
|  |  | Synovial WBC | | 0.90 (NR) | 2,315 cells/mL | 81.0 (NR) | 96.0 (NR) |  |
|  |  | Synovial PMN% | | 0.88 (NR) | 56.80% | 77.0 (NR) | 96.0 (NR) |  |
| Sebastian et al. 2 2025 [31] | Hip  chronic >90 d | Absolute PMN count (APMN) | EBJIS | 0.92 (0.88–0.96) | 783.6 cells/µL | 86 (NR) | 92 (NR) | EBJIS definition  616 revisions (177 hip, 439 knee)  Synovial WBC and PMN% on Sysmex XN‑550  Same-encounter testing  ROC excludes EBJIS “infection likely” and metallosis/polyethylene wear |
|  |  |  | ICM 2018 | 0.93 (0.89–0.97) | 783.6 cells/µL | 87 (NR) | 94 (NR) | ICM 2018 |
|  |  | Synovial WBC | EBJIS | 0.91 (0.87–0.96) | 1,630 cells/µL | 87 (NR) | 90 (NR) | EBJIS definition  Automated cell count on Sysmex XN‑550 |
|  |  |  | ICM 2018 | 0.92 (0.88–0.97) | 1,630 cells/µL | 88 (NR) | 94 (NR) | ICM 2018 |
|  |  | Synovial PMN% | EBJIS | 0.88 (0.83–0.93) | 65.90% | 73 (NR) | 92 (NR) | EBJIS definition  Differential on Sysmex XN‑550 |
|  |  |  | ICM 2018 | 0.89 (0.84–0.94) | 65.90% | 75 (NR) | 94 (NR) | ICM 2018 |
|  |  | α‑defensin (quantitative) | EBJIS | 0.84 (0.74–0.95) | 0.75 (index units) | 72 (NR) | 100 (NR) | EBJIS definition  Quantitative α-defensin on frozen aliquots Head‑to‑head with APMN/WBC/PMN% |
|  |  |  | ICM 2018 | 0.82 (0.71–0.94) | 1.20 (index units) | 69 (NR) | 100 (NR) | ICM 2018 |
|  | Knee  chronic >90 d | Absolute PMN count (APMN) | EBJIS | 0.91 (0.88–0.94) | 549 cells/µL | 83 (NR) | 90 (NR) | EBJIS |
|  |  |  | ICM 2018 | 0.91 (0.88–0.94) | 594.2 cells/µL | 83 (NR) | 92 (NR) | ICM 2018 |
|  |  | Synovial WBC | EBJIS | 0.90 (0.86–0.93) | 1,910 cells/µL | 78 (NR) | 96 (NR) | EBJIS |
|  |  |  | ICM 2018 | 0.90 (0.86–0.93) | 1,910 cells/µL | 78 (NR) | 95 (NR) | ICM 2018 |
|  |  | Synovial PMN% | EBJIS | 0.89 (0.85–0.92) | 54.30% | 79 (NR) | 93 (NR) | EBJIS |
|  |  |  | ICM 2018 | 0.90 (0.86–0.93) | 54.30% | 80 (NR) | 92 (NR) | ICM 2018 |
|  |  | α‑defensin (quantitative) | EBJIS | 0.82 (0.77–0.88) | 0.40 (index units) | 66 (NR) | 98 (NR) | EBJIS |
|  |  |  | ICM 2018 | 0.82 (0.77–0.88) | 0.40 (index units) | 68 (NR) | 95 (NR) | ICM 2018 |
| Shi et al. 2022 [24] | Female | D-dimer | | 0.79 (0.705–0.879) | 605 ng/mL | 65.9 (NR) | 79.7 (NR) | — |
|  |  | CRP | | 0.96 (0.914–1.000) | 8.86 mg/L | 90.2 (NR) | 95.7 (NR) |  |
|  |  | Fibrinogen | | 0.89 (0.826–0.953) | 3.54 g/L | 85.4 (NR) | 84.1 (NR) |  |
|  |  | ESR | | 0.93 (0.882–0.976) | 27.50 mm/h | 87.8 (NR) | 85.5 (NR) |  |
|  |  | CRP/Albumin | | 0.96 (0.914–1.000) | 0.22 (unitless) | 90.2 (NR) | 94.2 (NR) |  |
|  | Male | D-dimer | | 0.76 (0.662–0.862) | 1,070 ng/mL | 46.2 (NR) | 97.0 (NR) |  |
|  |  | CRP | | 0.91 (0.867–0.973) | 10.64 mg/L | 76.9 (NR) | 91.0 (NR) |  |
|  |  | Fibrinogen | | 0.87 (0.799–0.937) | 3.80 g/L | 76.9 (NR) | 85.1 (NR) |  |
|  |  | ESR | | 0.92 (0.874–0.974) | 14.50 mm/h | 84.6 (NR) | 86.6 (NR) |  |
|  |  | CRP/Albumin | | 0.91 (0.867–0.973) | 0.31 (unitless) | 82.1 (NR) | 92.5 (NR) |  |
| Suren et al. 2023 [38] | | CP (LFT) – all implants | | 0.94 (0.89–0.99) | Qualitative/continuous | 94 (80–99) | 87 (79–93) | Compared with pre‑op ICM score (Se 0.71; Sp 1.0) |
|  |  | CP (LFT) – revision/tumor | | 0.92 (0.82–1.0) | Qualitative/continuous | 89 (67–99) | 80 (65–90) |  |
| Theil et al. 2024 [32] | | Synovial WBC | | 0.916 (0.862–0.970) | 1,200 cells/μL | 94.5 (NR) | 75.5 (NR) | EBJIS reference thresholds (1,500 cells/μL; 65% PMN) |
|  |  | Synovial PMN% | | 0.821 (0.739–0.902) | 63% | 85.5 (NR) | 73.6 (NR) |  |
| Wang et al. 2021 [42] | | CRP (serum) | | 0.703 (0.601–0.791) | 10.2 mg/L | 84.6 (69.5–94.1) | 56.9 (43.2–69.8) | MSIS 2013  Only chronic PJI  THA+TKA |
|  |  | Synovial PMN% | | 0.810 (NR) | 69.79% | 84.62 (NR) | 74.14 (NR) |  |
|  |  | Synovial CRP | | 0.937 (NR) | 7.26 mg/L | 84.62 (NR) | 93.10 (NR) |  |
| Wixted et al. 2023 [25] | | D-dimer | | NR | >860 ng/mL | 93.5 (NR) | NR | RS in paper:  CRP >1 mg/dL  ESR >30 or >50 mm/h  D‑dimer >860 ng/mL |
| Xu et al. 2022 [26] | | CRP (serum) | | 0.882 (NR) | 7.39 mg/L | 79.1 (NR) | 86.0 (NR) | ICM 2013  Histology >5 PMN/HPF in 5 fields |
|  |  | ESR (serum) | | 0.809 (NR) | 42.5 mm/h | 65.5 (NR) | 84.2 (NR) |  |
|  |  | Fibrinogen (serum) | | 0.834 (NR) | 3.67 g/L | 69.6 (NR) | 86.5 (NR) |  |
|  |  | IL‑6 (serum) | | 0.845 (NR) | 8.59 pg/mL | 70.9 (NR) | 86.5 (NR) |  |
|  |  | PLT | | 0.684 (NR) | 201.5 × 10^9^/L | 61.5 (NR) | 69.8 (NR) |  |
|  |  | MLR | | 0.686 (NR) | 0.30 (unitless) | 54.1 (NR) | 79.1 (NR) |  |
|  |  | NLR | | 0.659 (NR) | 2.90 (unitless) | 62.8 (NR) | 66.5 (NR) |  |
|  |  | PLR | | 0.674 (NR) | 126.11 (unitless) | 72.3 (NR) | 57.1 (NR) |  |

**Abbreviations:** AUC=area under the receiver operating characteristic curve; CI=Confidence interval; CRP=C-reactive protein; CP=Calprotectin; NR=Not reported; MSIS=Musculoskeletal Infection Society; PJI=Periprosthetic joint infection; ICM=International Consensus Meeting; ESR=erythrocyte sedimentation rate; WBC=White blood cell count; PMN=polymorphonuclear neutrophils; LE=Leukocyte esterase; ELISA=Enzyme-linked immunosorbent assay; MTP=Metagenomic microbiome profiling; LFT=Lateral flow test; pos=positive; neg=negative; THA=total hip arthroplasty; TKA=total knee arthroplasty; PCT=Procalcitonin; FDP=fibrin degradation product; PLT=platelet count; PVR=platelet volume ratio; IDSA—Infectious Diseases Society of America; Se=Sensitivity; Sp=Specificity; intra-op=intra-operative; NGAL=Neutrophil gelatinase-associated lipocalin; pre-op=pre-operative; EBJIS=European Bone and Joint Infection Society; IL=Interleukin; RA=Rheumatoid arthritis; MLR=monocyte-to-lymphocyte ratio; NLR=neutrophil-to-lymphocyte ratio; PLR=platelet-to-lymphocyte ratio.

**Reconstruction of 2×2 tables for diagnostic accuracy analyses**

For each study and index biomarker included in the quantitative synthesis, we extracted true positives (TP), false positives (FP), false negatives (FN), and true negatives (TN) whenever these data were directly reported. When raw 2 × 2 counts were unavailable, they were reconstructed from the reported sensitivity and specificity using the numbers of infected and non-infected participants in the same analytic cohort/subgroup and for the same test. Specifically, TP and TN were estimated from sensitivity × diseased sample size and specificity × non-diseased sample size, respectively, and FN and FP were obtained by subtraction.

When sensitivity and specificity were reported as rounded values, integer cell counts were selected to reproduce the published estimates at the reported level of precision. If more than one integer solution was possible because of rounding, we retained the solution most consistent with all other available study-level information, including subgroup sample size and, where available, other diagnostic parameters (e.g., PPV, NPV, overall accuracy, or 95% confidence intervals).

Test-specific denominators were used when not all participants had evaluable results for a given index test. Intermediate/indeterminate index-test results and inconclusive reference-standard classifications were handled according to the rules used in the original study; otherwise, only clearly classifiable infected and non-infected cases were included. A study-level supplementary table reports TP, FP, FN, and TN for each included study/test and indicates whether counts were directly reported, derived from reported frequencies, or reconstructed.

**Table S4.** Study-level 2 × 2 tables used in the quantitative synthesis, indicating whether counts were directly derived from reported subgroup frequencies or reconstructed from published diagnostic accuracy estimates.

| **Study ID** | **Subgroup included in meta-analysis** | **Biomarker** | **Cutoff / Platform** | **Infected**  **N** | **Non-infected**  **N** | **TP** | **FN** | **FP** | **TN** | **Source of 2 × 2 counts** |  |
| --- | --- | --- | --- | --- | --- | --- | --- | --- | --- | --- | --- |
| **Chronic PJI (>= 90 d): pooled accuracy by test (k <= 3) with individual studies** | | | | | | | | | | | |
| Baek 2023 | Knee | Synovial PMN% | Not reported | 18 | 14 | 16 | 2 | 1 | 13 | R |  |
| Baker 2022 | Overall (collapsed hip + knee subgroup counts) | Synovial PMN% | 80% | 194 | 394 | 168 | 26 | 3 | 391 | D |  |
| Sebastian 2025 | Chronic ≥90 d subset | Synovial PMN% | Not reported in supplied dataset | 119 | 207 | 110 | 9 | 31 | 176 | R |  |
| Baek 2023 | Knee | Synovial WBC | Not reported | 18 | 19 | 17 | 1 | 0 | 19 | R |  |
| Baker 2022 | Overall (collapsed hip + knee subgroup counts) | Synovial WBC | 3,000/µL | 194 | 394 | 177 | 17 | 6 | 388 | D |  |
| Sebastian 2025 | Chronic ≥90 d subset | Synovial WBC | Not reported in supplied dataset | 123 | 203 | 115 | 8 | 26 | 177 | R |  |
| **Chronic PJI (>= 90 d): tests with two studies (no pooling**) | | | | | | | | | | | |
| Baker 2022 | Overall (collapsed hip + knee subgroup counts) | Serum CRP | 1.0 mg/dL | 194 | 394 | 162 | 32 | 46 | 348 | D |  |
| Sebastian 2025 | Chronic ≥90 d subset | Serum CRP | Not reported in supplied dataset | 122 | 204 | 113 | 9 | 28 | 176 | R |  |
| Baker 2022 | Overall (collapsed hip + knee subgroup counts) | Synovial CRP | 6.9 mg/L | 194 | 394 | 145 | 49 | 8 | 386 | D |  |
| Sebastian 2025 | Chronic ≥90 d subset | Synovial CRP | Not reported in supplied dataset | 133 | 193 | 116 | 17 | 25 | 168 | R |  |
| **Chronic PJI (>= 90 d): tests with a single study (no pooling)** | | | | | | | | | | | |
| Baek 2023 | Knee | Alpha-defensin ELISA | 1,580 µg/L (ELISA) | 18 | 19 | 17 | 1 | 2 | 17 | R |  |
| Baker 2022 | Overall (collapsed hip + knee subgroup counts) | Alpha-defensin LFT | LF/IA | 194 | 394 | 190 | 4 | 50 | 344 | D |  |
| Ackmann 2022 | Overall | Serum calprotectin | 9,910 ng/mL (immunoturbidimetric assay) | 26 | 55 | 21 | 5 | 5 | 50 | D |  |
| Suren 2023 | Overall | Calprotectin LFT | Lyfstone LFT (mid risk = negative) | 50 | 87 | 47 | 3 | 11 | 76 | R |  |
| Baek 2023 | Knee | Leukocyte esterase | 2+/3+ positive | 16 | 19 | 6 | 10 | 0 | 19 | R |  |
| Baker 2022 | Overall (collapsed hip + knee subgroup counts) | Serum ESR | 30 mm/h | 194 | 394 | 170 | 24 | 107 | 287 | D |  |

**Abbreviations:** TP, true positive; FN, false negative; FP, false positive; TN, true negative; CRP, C-reactive protein; ESR, erythrocyte sedimentation rate; PMN, polymorphonuclear leukocyte; LFT, lateral flow test; ELISA, enzyme-linked immunosorbent assay; R: Reconstructed counts were obtained from published sensitivity/specificity and the corresponding infected/non-infected denominators of the same analytic subgroup. D: Derived from reported subgroup counts, which were obtained by summing hip and knee subgroup-specific 2×2 tables within the same study after excluding duplicated overall rows, to avoid double-counting in the meta-analytic dataset. Test-specific denominators were used when not all patients had evaluable results for a given biomarker. Therefore, infected/non-infected totals may differ across tests within the same study.
